# Spirit of the Foreign Periodicals, &c.

**Published:** 1841-10-01

**Authors:** 


### Spirit of the Foreign Periodicals, &c.

### M. Forget's Letter to M. Andral on Modern Humorism.

The readers of the two last Numbers of the *Medico-Chirurgical Review* have probably perused the copious extracts which we have given (in the Foreign Periscope) of the lectures recently delivered by M. *Andral* on the changes of the blood in various diseases. Coming from so high an authority on pathological subjects, they could not fail to attract much notice from his countrymen, who, it is well known, have been so long, and still are, deeply imbued with the doctrines of *Broussais* and his disciples.

Fortunately for the progress of practical medicine in *this* country, these doctrines have never obtained so much favour, nor influenced to the same extent the minds of the profession here as on the Continent; we say *fortunately*, because we believe that nothing has more contributed to vitiate the practical value of most French medical writings than the all-pervading spirit of what has been most absurdly called the "Physiological System." That any system should be called physiological, which confines itself to the study of the *solids* of the body, and totally excludes all consideration of the *fluids*, is surely a very strange misapplication of language; but such is the influence of dogmatism in medicine that we do not remember having ever met with any exposure of the fallacy in a single work which has, during the last ten or fifteen years, issued from the French press.

On numerous occasions, while commenting on the selections from foreign periodicals in this Journal, have we alluded to this strange error of our continental brethren; and it is therefore probable that few of our readers have allowed their minds to be deluded by it. Indeed there is something so essentially practical in the character of Englishmen, that any mere dogma, however ingenious, and however earnestly it may be insisted upon, is not likely to prevail with them for any length of time, if its precepts are not found to be consonant with the results of experience. The doctrine of the inflammatory origin of almost all diseases, as taught by the so-called physiological school, was therefore not likely to gain many adherents in this country, and in the present day the term of *gastro-enterite* is, on the whole, rarely met with in our medical literature.

If this were a topic of merely speculative interest, it would not be worth while to occupy the attention of the public with it; but it cannot be so: for the treatment of diseases will necessarily always be more or less influenced by, if not actually based upon, the opinions that are formed of their proximate nature and of their origin. Now there is no class of diseases on which it is more important to have rational ideas than that of fevers. They are in their very character, the mode of their development, the varying features they present, their wide-spreading extension, their repugnance to curative means, their often fearful devastation, and the inconstant and unsatisfactory traces of their action left behind in the dead body, the most puzzling of all the maladies to which flesh is heir to; and unfortunately it seems but too probable that they will remain so, unless indeed we can discover some other means of detection and close-prying observation than we at present possess, or discover some new chemical agents which can control and resist them. That they arise from the operation of some miasmatic —(we are aware that the meaning of the word is most ambiguous)—influence, which acts as a poison on every part of the human economy, cannot, we think, be well disputed; and we have thus been always led *à priori* to infer that their chief, if not their primary, action is on the fluids of the body. We have therefore hailed with much pleasure the efforts recently made by M. *Andral* and other good observers, both in France and in this country, to discover the changes which the various fluids undergo in fevers as well as in other diseases.

The following letters will perhaps sufficiently explain themselves; we could indeed have wished that that of M. *Forget* had been more perspicuous; but as it is, we give most of its leading paragraphs literally translated.

My dear and much honoured Master,

It was a practice among the learned men of former times to address each other in familiar letters, in which they mutually suggested and discussed the leading questions of professional interest that happened to arise at the time. It is to this practice that we are indebted for the beautiful answers of *Sydenham* to *Robert Brady, Henry Paman,* and *William Cole,* on the medical constitutions of the seasons, and on the venereal disease, smallpox, and hysteria: and also for those valuable replies, full of medical wisdom, addressed by *Torti* to *Muratori.* No method, it seems to me, can be more favourable to the development of our ideas than this epistolary correspondence; for, while free scope is given to the expression of our thoughts, the veneration and respect due to certain men restrain us within the bounds of courtesy and gentlemanly propriety. In the arena of science, as well as in the saloons of fashionable life, is it not becoming that well-born men should always address each other with politeness (chapeau bas)? and moreover is it not much more easy to understand each other when thus mutually courteous, than under the double irritative influence of anger and contradiction, feelings which so often give rise to those furtive, and consequently often most unbecoming personal attacks, so common in the pages of our medical journals?

Be pleased therefore to accept with favour this address of one of your pupils, who has become in his turn a teacher of others, and one whom his position calls upon to explain every subject connected with a science that is entrusted to his teaching. A minister of this religion, whose object is the life of our fellow men and whose symbol is humanity, speaking in the face of an enlightened public to a master whom I love and venerate, my pen can surely give expression to nothing save the most cordial convictions of profound respect and admiration.

...... When we see the most able and candid observers successively sapping the fundamental principles of their predecessors; when we see a new chemical or microscopical analysis overturning the results of former enquiries; when we see authors differing from each other even as to the physical qualities, such as the colour and consistence, of the blood, is it not natural and at the same time prudent to admit with reserve the recently announced discoveries on the humoral conditions of this fluid in different classes of diseases?

...... A most important result of your researches is the nosological inference that, while genuine inflammatory diseases are characterised by an *excess* of fibrine in the blood, there is a *diminution* of this constituent in fevers. If this conclusion be found to be accurate, you have achieved a most Herculean task; for with a strong hand you have sealed the long disputed traditions of antiquity, and at the same time you have cut short the interminable discussions of modern essentialists and localisers. You have with one stroke deified Galen, and utterly overthrown Broussais.......

Certain difficulties have presented themselves to my mind in the consideration of some of the results of your researches: these I will now submit to your attention.

You state, "in the inflammations called primitive, as in pneumonia, the fibrine of the blood is in excess." What will be said to this assertion by those gentlemen, like M. *Cruveilhier* and many self-called Hippocratists, who consider every inflammation as secondary, and tell us that pneumonia is just as much secondary in its nature as variola or follicular enteritis? You will no doubt reply to this by appealing to the difference in the quantity of the fibrine of the blood in these diseases, a difference which, in your opinion, is sufficient to demonstrate that this nosological fusion is radically wrong. Be it so; it is still a subject of dispute.

Again, you state:

"In fevers (exanthemata, typhus) the proportion of the fibrine is, in the greater number of cases at least, diminished—unless," you add, "there exists an intercurrent inflammation, which immediately raises the quantity of the fibrine." Now here, in my opinion, our real difficulties commence. And first, we may suggest, it would be very desirable that this diminution were met with in all simple cases; otherwise, advantage will be taken of the exceptions to contest the truth of the principle. Then again, if the fibrine resumes its excess, whenever a concomitant inflammation makes its appearance, may we not infer that the opposite condition of the blood—that wherein the quantity of the fibrine is below the healthy standard—must be the exception and not the general rule? For is there not a concomitant inflammation in every eruptive fever? unless indeed we are to admit that the pustules of smallpox, the *dermitis* and angina in scarlet fever, and the *dermitis* and bronchitis in measles, are not genuine inflammation. But, pray, what is a morbid action characterised, as the smallpox pustule is at its different stages, by redness, heat, swelling and suppuration, if it is not inflammation? Perhaps, indeed, it may be objected that such inflammations are of a secondary nature, or of a specific character; but pray what are we to understand by such subtle distinctions?

Now as I am fully persuaded that follicular enteritis is almost invariably present in typhus, and as this enteritis cannot be regarded otherwise than as a concomitant inflammation, we might surely expect to find that the quantity of the fibrine in the blood is increased rather than diminished in the course of the disease,—since, according to your own principles, the occurrence of inflammation during a fever is always attended with such a condition of the circulating fluid. For I must confess to you that follicular enteritis is in my eyes an inflammation, until some one has shewn me that it is something else, and that redness, swelling, heat, ulceration and suppuration combined, are not the inherent characters of this morbid action.

Another important and fundamental question, on which, if I mistake not, you have not been sufficiently explicit, is whether the diminution of the fibrine in eruptive and typhoid fevers is initial and primitive, or consecutive and accessory, or whether it be a preceding lesion. This seems to me most essential to determine; for if the defect of the fibrine be primitive, if it be the germ of the disease, the great therapeutic indication in its treatment must be to destroy and eradicate this germ. If such be the case, must we not consider that *Sydenham* has been wrong in the principles which he has laid down for the management of febrile disorders? and that the *Brunonian* practice is greatly to be preferred to that recommended by *Broussais?*

Now though ordinary practice has proved the superiority of antiphlogistic treatment in the cure of these diseases in general, I admit that this circumstance is not in itself sufficient to overturn the alleged fundamental fact of the deficiency of the fibrine; for it must be allowed that there are not a few facts in medicine which, although we cannot explain, we must not refuse our assent to; but still it will surely be acknowledged that the circumstance cannot fail to startle us, and make us somewhat distrustful of the discoveries you have announced.

But if, on the contrary, the deficiency of the fibrine be secondary, and consecutive to some other morbid element of an earlier and more constant occurrence —as the follicular enteritis seems to me to be—then your discovery loses much of its value at least in a practical and therapeutic point of view; for my own part I should have no reluctance to admit it; all that I have pretended to demonstrate being, that in follicular enteritis the alteration of the blood, were it more constant than it really is, is a secondary and not a fundamental phenomenon of the disease. As to the condition of the fibrine more especially, I have remarked (De l'Enterite Folliculeuse, p. 518) "that there is nothing after all surprising that a morbid state which vitiates the process of nutrition at its very source should cause a change in the *crasis* of the blood." Now as you speak of *typhoid* fevers, and as the typhoid state is at first by no means uniform, and far from being, in all cases, an initial phenomenon in follicular enteritis, it follows that even you perhaps may regard the diminution of the fibrine as only a secondary phenomenon. You see, my much honoured master, that a word on your part on these important matters is not less necessary than desirable.

But I am still more anxious to learn your opinion as to the therapeutic indications to be derived from your humoral discoveries of the state of the blood in typhus and other fevers. Do, therefore, inform us whether the deficiency of the fibrine requires, or not, a tonic and nutritive regimen? or whether it is compatible with the lowering system which is generally adopted in the present day?

There are several other points on which I long to hear your sentiments; but these must be deferred at the present time. May I, in conclusion, solicit an answer to the following three questions?

1. Is the diminution of the fibrine of the blood the most constant element of serious (graves) fevers? is it, for example, more constant than follicular enteritis?

2. Is the diminution of the fibrine primitive? or, at least, is it anterior to the follicular enteritis?

3. Does the diminution of the fibrine involve some fundamental modifications in the treatment of the disease? and are we to prefer a tonic to an antiphlogistic regimen?—*Gazette Medicale.*

### M. ANDRAL'S ANSWER.

My dear and honoured Confrere,

Your letter has warmly excited my interest, since I must suppose that the doubts, which have suggested themselves to a mind like yours as to the value of the results which my researches on the state of the blood have led me to, have occurred to many other medical men, and I thank you for giving me an opportunity of endeavouring to explain them. I shall do this the more willingly as it is to you, my dear confrere, whose character and talents I so much esteem, that my reply is addressed. I perfectly concur with you in the sentiment expressed in the commencement of your letter. You regret, and I also regret, the want of those scientific correspondences, of which our illustrious predecessors have given us many examples, and wherein they have joined in discussion on professional subjects with no little benefit to the advancement of science. I congratulate you in having recalled these recollections, and I trust that your letter and my answer may be also somewhat useful to that noble science to which we have both devoted our entire lives.

There are two parts in your letter; in the one you suggest certain objections to the method which I, in union with my excellent fellow-labourer M. *Gavarret*, have pursued to ascertain the morbid alterations of the blood; and in the other you attack some of the particular results which we have obtained. I will now reply to each of these separately.

No one in the present day disputes that, in the order of physiological as of pathological facts, the study of the fluids of the body deserves as much attention as that of the solids. The part which the alterations of the blood may have in the production of disease cannot in our time be a subject of disagreement. But there is still considerable difference of opinion as to the best method to be followed with the view of introducing some precision into the study of these alterations. Permit me therefore to converse with you a little on this subject.

As long as we content ourselves with trying to establish the reality of these alterations by mere reasoning, whether by dwelling on the functions of the blood in health, and on the reciprocal influences of this vital fluid and of the solids on each other, or by attending to certain causes and certain symptoms of diseases, we can never arrive at any certainties of knowledge. Of late years another plan has been pursued. By modifying the blood in a variety of ways in different animals, M. *Majendie* has induced in them a certain number of morbid states more or less closely resembling those which arise spontaneously in man, and a legitimate analogy has led him to infer that in the case of mankind the starting point *(point de depart)* of several diseases is to be found in one or other of those modifications of the circulating fluid which he had artificially induced in the lower animals. But to demonstrate that such is really the case, we require to advance another step. In the place of induction, we must substitute direct experimentation; we must examine the blood in man under the influence of disease, and thus try to ascertain if it really has undergone any decided change. Now by acting in this way, we soon discover that the blood does become changed in a variety of ways; and thus, very fortunately for science, different modes of experimenting mutually concur to the establishment of the truth. For while, on the one hand, M. *Majendie*, by withdrawing a portion of the fibrine from the blood of animals, induced in them various congestions and hæmorrhages, M. *Gavarret* and I have ascertained that the blood of a scorbutic patient, whose skin was covered with large ecchymoses, had not more than a third part of its normal quantity of fibrine: and we have proved that the tendency to hæmorrhages is just in proportion to the amount of the deficiency of this element in the blood.*

You, my dear *confrere*, are not willing to admit the truth of these results without reserve. It would not become me, who have passed my life in proclaiming the necessity of *doubting* upon all occasions, to be surprised that you should hesitate to give your assent all at once. The sentiment I allude to is well expressed by Haller:—*noviter repertarum rerum neque injustum fatum est, ut serius aliquanto fidem impetrent. Prestiterit enim aliquantum in dubitationis partes declinasse, quam novas neque satis firmas undique opiniones, absque judicio aut examine, recipere.*

The method which I have followed in the study of the changes of the blood in disease is, while availing myself of all chemical discoveries, carefully to examine those alterations which occur in the composition of this fluid, and not be satisfied with merely observing the more striking physical phenomena,—such as the formation of a buffy coat, its thickness, the consistence of the coagulum, the quantity of serum—such as is usually done. Not that I at all undervalue the importance of watching these latter very attentively; they communicate most valuable information; but then this information is sometimes inexact, and more often still, it is imperfect.

I have shewn elsewhere that changes in the mere aspect and outward appearance of the blood are not uniformly attended with corresponding changes in its composition. It becomes therefore necessary to separate, by means of certain processes which are well known to physiologists, the different constituents of the blood from each other, and thus to ascertain the changes in the relative proportions of each of these constituents. To proceed in this manner seems to me to be only applying to the study of the changes of the blood the same method of

---

* This position can scarcely be admitted as a general or absolute principle or axiom, without at least keeping in mind at the same time the distinction between active and passive hæmorrhages. Every one knows that the blood drawn in many cases of hæmoptysis is apt to be buffy, and that this form of hæmorrhage is most frequent in persons disposed to or affected with phthisis—a disease in which, according to M. *Andral*, there is usually an excess of fibrine in the blood. —*(Rev.)*

investigation that has been pursued with so much success in the examination of the solids of the body. Long ago *Silvius de la Boe* very justly remarked that anatomy was a sort of chemistry, and chemistry in its turn was a sort of anatomy; "for," said the illustrious Hollander, "in both studies our object is to separate and reduce to their elements compound bodies, the blood, for example, to its principles, and any organ to the tissues which compose it." This is quite true; we must analyse the solids by anatomical and the fluids by chemical examination. The first of these studies is far advanced, but the latter is still very imperfect. It seems to me that in the present day we are nearly about the same stage in our progress to the knowledge of the humoral changes that *Hippocrates* was in reference to the changes of the solids: in his works he points out as possible, rather than authoritatively enunciates, the existence of diseases in the form or the relations of the solids. Now this is nearly our condition in reference to the blood. We have stopped with the observation of certain modifications of its external aspect, as the old Coan did with the description of some external phenomena of the solids.

...... It will not be suspected that I wish to return to the fanciful doctrines of *Paracelsus* or of *Boerhaave;* I do not wish to see medicine exclusively tributary to chemistry more than to anatomy: the aid of both sciences contemporaneously is necessary to advance its real progress.

In some cases the material element of disease exists exclusively in the blood; in others, in the solids; and in a third set it is present in both at the same time. For example, I, for my part, am of opinion that pneumonia cannot exist without an increase in the quantity of the fibrine of the blood, as well as without some of those morbid alterations of structure which characterise one or other of the three stages of the disease; and, if we may be permitted to judge of the importance of any phenomenon by its constancy, not a little value will be attached to the change in the proportion of the fibrine in the present instance; for out of 76 analyses made by me of the blood from pneumonic patients, there was not one in which an excess of the fibrine was not detected.

But you say, how can we trust to the results of examination of the fluids, when we find them stated so discordantly by different writers? Observe, my dear *confrere,* that the same reproach may be made to nearly every branch of science. Some have denied the utility of morbid anatomy, because it also has its contradictory facts, or its discrepant interpretations of the same phenomena. The value of medicine itself has been questioned, because it is so fluctuating and variable. But in spite of the difficulty of collecting accurate facts, and of interpreting them aright, we may be assured that every science is progressively advancing, however slow and unsteady the steps may be; and that, in proportion as it follows the necessary law of its development, the errors which retarded it in its course, are successively recognised and dissipated, while the truths which it has discovered become every day more manifest and clear.

Organic chemistry is still an infant science; it would be unfair, therefore, to distrust its actual and positive results, because they have almost nothing analogous with those which were formerly obtained, when physicians knew of no other method of analysing the blood, except by macerating, fermenting, and distilling it in a variety of ways.

It moreover seems to me, my dear sir, that you have exaggerated to yourself the number and the importance of the contradictory statements in modern works on the blood. In this part of your letter you cite some names, and you seem disposed to predict that, one of these days, I shall be setting myself to replace MM. *Denys, Lecanu,* and *Mandl* in the very rank from which I have tried to displace them. There is surely some mistake here; for I have the very highest opinion of the value of the researches of these gentlemen. In many respects, I have taken their researches as the starting point for my own; and the numerous experiments which M. *Gavarret* and myself have made, have fully confirmed the accuracy of many of the details recorded in the work of M. *Lecanu*, and have not contradicted one of his general principles. He has not studied the fibrine apart and separated from the other elements of the blood, whereas on the contrary, we have always done so; but this is only a progressive step, and cannot be considered as a contradiction.

Again, in reference to M. *Denys*, whose interesting researches on the blood have given him a high rank among scientific men, I may say that there is no discordancy between his views and mine; only we have not been working in the same field. One of the leading ideas in M. *Denys*' work is to prove that albumen and fibrine are identically of the same nature; now I do not deny the identity; for some of the most distinguished chemists of the present day admit it, and M. *Liebig* has recently confirmed their views.

But whatever may be the ultimate opinion of chemists on this subject, it will remain no less true that, by stirring or switching fresh-drawn blood we can separate a particular element or constituent, which, although it may be essentially of the same nature as that which remains in solution or suspension in the remaining fluid, must in a physiological and medical point of view at least be very carefully distinguished.

This element, which we know by the name of fibrine, is specific in its functions as well as in its character; and the increase or diminution of its quantity present in the blood characterises in a very remarkable manner certain classes of diseases. I have therefore nothing to find fault with in the work of M. *Denys*; it is to be considered in one point of view, and mine in another. It is thus, that in the science of general anatomy physiologists have endeavoured to prove that all the tissues of the body, with the exception of the muscular and the nervous, are only simple modifications of the cellular substance, to the state of which they may, by particular processes, be reduced. But it still remains no less true that this idea, however correct it may be in the eyes of the mere anatomist, will not satisfy the physiologist and the physician. In their eyes, the fibrous tissue, the serous tissue, and the mucous tissue, although all reducible to the state of cellular substance, are distinct and separate tissues, because each of them has a secondary structure which is peculiar to it, and which adapts it for the performance of its specific functions.

I come now to the second part of your letter, in which you ask of me explanations of certain difficulties, and state certain objections to the practical value that is to be attached to the changes which the blood may exhibit in typhus fever.

I have said, and I repeat here, that those diseases, which have been long known by the name *Phlegmasiæ*, are distinctly separate from those which are usually called *Pyrexiæ*, and that they are distinguishable from each other by the different conditions of the blood that may be discovered on an analytic examination of this fluid.

In the acute *Phlegmasiæ* the fibrine of the blood is uniformly increased in quantity; nothing is more common than to find it double or triple its normal proportion. This increase of the fibrine we have ascertained to be present from the very commencement of the inflammatory attack. I am not yet in possession of any facts to shew that this augmentation takes place before the local morbid process is developed in some structure or another; on the contrary, in several cases where I have had the opportunity of examining the blood of a patient on the day before the attack, as well as on the day on which it took place, M. *Gavarret* and myself have assured ourselves that in the former case there was no increase in the quantity of the fibrine.*

* This announcement should be received with considerable reservation. For our own part, we are rather inclined to believe that the very contrary is the case.

In the *Pyrexiæ*, or proper fevers, the proportion of the fibrine in the blood is found to be either not affected at all, or it is less than in health; but it is never augmented—an important distinction between this class of diseases and the genuine *Phlegmasiæ*. When, indeed, the fever is not primary, but only the symptom or consequence of the inflammatory action, then the condition of the blood is regulated by the phlegmasia, and exhibits the lesion which is proper to it. We must here observe that a fever may be attended with two sorts of inflammation: 1. There may be an inflammation which has its origin or starting point in the common cause of the fever itself; as for example the cutaneous affection of the exanthemata; in this case the local lesion is connected with a cause that is more general. The blood remains the same as before the cutaneous inflammation was developed, and the local disease does not affect its constitution; hence the proportion of its fibrine is not increased.* 2. Things are, however, very different in those cases of fever in which the inflammation exists as a complication, such as pneumonia, bronchitis, &c.; for then the blood exhibits the characters that are proper to the phlegmasiæ, and the proportion of the fibrine is therefore found to be higher than in health.

Of 52 bleedings practised on 21 patients affected with typhoid fever, in one case only was the proportion of the fibrine of the blood found to be increased; and in that case there was a distinct pneumonia present: in all the other cases the proportion of this element was either normal or it was below the natural standard.

The highest proportion was 3½; and the lowest was 0.9;—the least quantity of fibrine which we have ever met with in any disease. As might be expected, this notable diminution of the fibrine is observed in the *adynamic* forms of the fever; the prostration of the vital powers being usually proportionate to the amount of diminution. During convalescence the quantity of the fibrine is found gradually to rise.

So much for the changes of the fibrine in the Phlegmasiæ and in the Pyrexiæ; let us now see how another constituent of the blood, the globules, are affected in these two classes of disease. As a general remark, we may state that the proportion of the globules is usually lower than in health, in the first, and higher than in health in the second class, at least during the early stages of the diseases. In estimating the proportion of this element of the blood in any case, it is most necessary to have our attention directed to the influence of the treatment and regimen which have been followed; for a single bleeding and low diet during even a day or two will be found to have a marked influence in lowering the proportion of the red globules.

As a general remark, it may be asserted that the proportion of the red globules is above the normal standard (127 in 1000 parts of blood) in *most*, but not in *all*, cases of fever at the commencement of the disesse, and before the use of any medical or dietetic means. The increase of the red globules in the Pyrexiæ is, however, neither so uniform nor so well-marked as the increase of the fibrine is in the Phlegmasiæ. In measles and in scarlatina I have found the proportion of the globules as high as 160 or 170.

There is a multitude of other topics which I should most willingly discuss

---

For several days, and sometimes weeks, before the *explosion* of an active phlegmasiæ, there are almost always certain precursory or prodromic symptoms, which emphatically indicate a plethoric state of the system, and an excess of over-rich blood in the vessels.—*Rev.*

* This points out a most important distinction between the morbid action in the genuine phlegmasiæ and that in the inflammatory affections of the exanthemata. The remedial inferences from this position are too obvious to require any illustration.—*Rev.*

with you, my dear confrere, but which the limits of a letter utterly preclude. That the question of the changes of the blood in disease is environed with numerous difficulties, is but too certain; but, if examined with due patience and research, we have reason to hope that its study will confer important service on practical medicine.—*Gazette Medicale.*

---

### M. Andral on the Changes of the Blood in Hæmorrhages.

The cause of certain hæmorrhages is to be found in a diminution of the normal proportion of the fibrine in the blood. This diminution may be either absolute, or only relative: thus 1, in some cases the fibrine alone is diminished, while the red globules of the blood remain in their normal proportion; and 2, the proportion of the fibrine is unaffected, but that of the globules is higher than in health. The former of these two conditions constitutes the *absolute,* and the latter the *relative* diminution of the element.

In a third set of cases the proportion of the fibrine seems to be diminished, while that of the globules is increased.

Now in every one of these three categories, the normal relative proportion between the two elements, the fibrine and the globules, is disturbed. The alteration may be either spontaneous, or it may be produced by sanguineous and other evacuations. In order that the proportion of the fibrine be diminished by the former, they must be carried to a considerable extent; their first and most marked effect being on the red globules, the proportion of which is very speedily diminished by any losses of blood. This diminution establishes a marked distinction between the hæmorrhagiæ and the phlegmasiæ, in the latter of which the proportion of the fibrine is found to be uniformly increased.

In seven cases of cerebral hæmorrhage, the blood was found to have a diminution of its proportion of fibrine, and an augmentation of that of its red globules. In one of these cases, the proportion of the fibrine had fallen to 1.9, while that of the globules had risen as high as 175; a few days afterwards the symptoms were more favourable, and a second venæsection was practised; the blood now drawn contained more than three parts of fibrine, and the proportion of the globules had fallen to 137—being only ten above the normal standard.

We have very little doubt, therefore, that many cases of cerebral hæmorrhage are owing to a diminution of the normal plasticity of the blood, in consequence of the diminution in the normal proportion of its fibrine.

*Cerebral Congestion.*—This is a morbid state which is as yet not at all well determined, and the anatomical lesions of which are by no means the same in all cases. Every symptom of the disease may be present; and yet in one case we find on dissection a highly injected state, and in another a remarkably anæmic paleness of the brain. In 15 cases of the disease, in which the blood was examined, the proportion of its fibrine was not, in a single instance, found to be increased, but in several it was certainly below the normal standard.

There is more than one point of anology between the states of typhoid fever and of cerebral congestion; in both diseases the precursory symptoms are much alike, and in both there is usually a diminution in the quantity of the fibrine of the blood.

*Albuminuria, or Morbus Brightii.*—M. *Andral's* experiments confirm the results of *Christison's* and *Rayer's* researches, as to the diminution of the quantity of the albumen in the serum of the blood, when this element, the albumen, is present in the urine.

*A Dissolved State of the Blood.*—The inorganic materials of the serum are—1, an alkali, and 2, neutral salts.

Much importance is attached by some pathologists to an increase in the proportion of the alkali, as the proximate cause of many diseases. It has been asked, for example, whether the proportion of the fibrine is not very much affected by the quantity of the free alkali in the blood: some facts seem to prove it. The quantity of alkali, we have reason to believe, is considerably increased in cases of scurvy, and of malignant typhus, in which the blood is known to be much less coagulable than it is in health. A mere excess in the proportion of the water of the blood is not, as is generally imagined, the cause of its being found to be in what has been called a *dissolved state,* as is abundantly proved by the phenomena which the blood in chlorosis is known to exhibit. It is rather the absolute or the relative diminution in the proportion of its alkaline ingredients, which constitutes the state of *dissolution* or abnormal fluidity of the sanguineous fluid.

*Presence of Pus in the Blood.*—Purulent matter has been found in the blood in the following cases: 1, in inflammation of the parietes of the heart, and of the bloodvessels; 2, in inflammation of other organs, as in smallpox, for example; in extensive abscesses, and then the matter must penetrate by absorption, molecule by molecule; 3, it has been met with in the middle of coagula, which had been formed during life, within the heart or large bloodvessels. The pus is found in different states; it may be infiltrated into the blood, so that it is conbined with this fluid, molecule with molecule, and then the blood exhibits a change in its colour; while in other cases it is deposited in the form of minute isolated drops in the midst of the blood, after death. M. *Piorry,* to whom we are indebted for some valuable researches on this topic, has observed on the surface and also in the substance of the buffy coat of the blood, drawn from patients labouring under pneumonia, white granulations, which he considers as particles of pus.

When purulent matter is mixed with the blood, a great change takes place in the constitution of the latter fluid; the coagulum loses its consistence, and becomes soft, friable, and reduced into small lumps; its liquid portion is a veritable ichor.

Other substances, the product of morbid action, are found, in certain cases, mixed with the blood. Thus cancerous and encephaloid matter has been frequently observed in the veins leading from and adjacent to parts which are the seat of these diseases.

The blood may also be infected by several poisons derived from the lower animals, as by that of glanders, of malignant pustule, &c. It has been found that the blood of over-driven animals, if injected into the veins of other beasts, produces serious accidents; the blood of the latter becoming soft, diffluent, and having little tendency to coagulate.—*Gazette Medicale.*

---

### M. ANDRAL'S RESEARCHES ON THE BUFFY COAT.

In one of the recent lectures, delivered last Summer at the Faculty of Medicine by this distinguished physician, we find the following statements, as to the relative frequency of the buffy coat in various diseases.

Of 12 cases of Acute *Amygdalitis,* there was a perfect buffy coat in 9, an imperfect one in 1, and none at all in 2—in 123 bleedings in cases of *Bronchitis,* the blood exhibited a perfect buff 35 times, an imperfect buff 25 times, and no appearance of it 63 times. In the cases in which the buff was perfect, there was acute capillary bronchitis; in the other cases, the disease was not accompanied with fever.—*Lead Colic:* of 10 bleedings, buff was found in 3 cases only.—*Chlo-*

*rosis:* of 11 bleedings, a perfect buff, similar to that observed in rheumatism, was found in 7 cases; once there was an imperfect buff, and in the remaining 3 cases there was none at all. We may suspect from these facts that the existence of the buff on the surface of the blood has not always the same signification, and that different morbid conditions may give rise to this phenomenon.—*Cerebral Congestion:* of 103 cases, a perfect buffy coat was found in 14 cases only; in 12 cases there was an imperfect one, and in the remaining 77 cases there was none at all. —*Pleuritic effusion:* some of the cases were chronic and apyretic, and others were recent and accompanied with slight fever; of 27 bleedings, the blood was buffy 7 times, slightly so 9 times, and not at all so 11 times.—*Intermittent Fever:* of 32 bleedings, a buff was observed in 5 cases only.—*Typhoid Fever:* of 187 bleedings, the blood was quite free from buff in 147 cases; in 30 there was an imperfect partial buff; and in 10 cases only, and these were complicated with pneumonia or bronchitis, was there a perfect buff. Surely therefore we may infer that, in simple uncomplicated typhoid fever, the blood is destitute of a buffy coat.—*Cerebral Hæmorrhage:* in 2 cases only out of 22 bleedings was the blood at all buffy.—*Hypertrophy of the Heart:* the blood was buffy in 11 cases only out of 72 bleedings.—*Albuminuria:* 6 cases; no buff found in any of them: Can we then regard the disease as nephritis?—*Pneumonia:* of 230 bleedings, a well-marked buff was observed in 215 of the cases; it was somewhat imperfect in the remaining 15.—*Acute Rheumatism:* of 134 cases, a perfect buff was found in 125; in 5 it was imperfect, and in 4 there was none at all. In one of these last cases the bleeding was practised towards the close of convalescence, when the proportion of the fibrine, which had been as high as *six*, had fallen down to *three*, nearly the normal standard.—*Chronic Rheumatism:* in 11 cases only, out of 50, was the blood found to be buffy.—*Measles:* 11 cases; no buff in any of them.—*Scarlet Fever:* 9 cases; no buff in any of them, except when there was acute nephritis co-existent.—*Pulmonary Tubercles:* 203 cases; the blood exhibited a perfect buff in 140, an imperfect one in 13, and none at all in 50 of the cases.

From the preceding data, M. *Andral* infers that the existence of the buffy coat does not necessarily indicate an absolute increase in the quantity of the fibrine in the blood, but only a relative increase of it in proportion to the quantity of the red globules.

When the blood contains an excess of its fibrinous element, the process of coagulation goes on more slowly than usual; thus the blood drawn from a person affected with acute rheumatism coagulates more slowly than the blood drawn from a typhoid patient. The globules are precipitated along with a small portion of the fibrine, the greater portion of this element being still in a state of solution in the upper stratum, and not coagulating until the globules have entirely subsided.

The increase of the relative proportion of the fibrine to the globules may occur in two different conditions:—1, the quantity of the globules being normal, that of the fibrine may rise from 3 or 4, the standard in health, to 10; under such circumstances the formation of the buffy coat is uniform and constant, its thickness and consistence being proportionate to the excess in the relative quantity of the fibrine. This is the case in all the genuine phlegmasiæ, in which there is a real and absolute increase of the fibrine. 2. The quantity of the fibrine may be normal, but that of the globules may be considerably reduced. Now in this case, although there is no absolute excess of the fibrine, a genuine and well-marked buffy coat, with retracted and puckered edges, may be formed on the blood, although there be no inflammatory disease present. Hence we observe this phenomenon in the blood of chlorotic girls. M. *Andral* has seen a perfect buff on the blood when the proportion of its globules had fallen from 127, the normal standard, to 28.

The explanation of these facts is of the greatest practical importance, and puts an end to those disputes which have taken place among medical men, who have been surprised to find a buffy coat on the blood in chlorosis—a disease which has usually been considered as quite antipodal to the phlegmasiæ.

The mere presence of the buffy coat is by no means an indication of the existence of inflammatory action; all that it indicates is that there is an alteration in the relative quantities of the fibrine and of the red globules, by which there is an excess, either absolute or relative, of the former element.

The last circumstance that we can allude to is, that we must not, in all cases, judge of the actual quantity of the fibrine and red globules by the size of the clot; for this depends much on the force with which it has contracted during the process of coagulation. Thus we not unfrequently observe a large coagulum, when the blood is really deficient in its solid materials, in consequence of a great portion of the serum being retained in its meshes. We should therefore, in all cases, pay great attention to the consistence, as well as to the size of the coagulum.—*Gazette Medicale.*

---

### M. VELPEAU: NEW APPLICATION TO ERYSIPELAS, WITH REMARKS ON OTHER REMEDIES.

M. *Velpeau* informs us that of late he has been using a solution of the sulphate of iron with most decided advantage, as an application to parts affected with erysipelas. According to his experience its effects are, in most cases, very rapidly useful, the inflammation usually subsiding in from 24 to 48 hours after it has been applied.

He talks of its seeming to have some specific action, and goes on to say: "we maintain that this topical remedy is the only one which, as far as we know, so quickly arrests erysipelatous phlegmasiæ." (!) Its action is strictly local and confined to the part to which it is applied; for he has often seen the erysipelas disappear in a part that was kept wetted with the solution, while the circumferential parts were more or less affected with it.

M. *Velpeau* then comments on the results which he has obtained from the use of other local remedies.

*Compression.*—His remarks are so pertinent in some respects, and so truthfully expose the senseless conduct of too many in our profession, that we shall give them:—

*Ignorant Abuse of Remedies.*—" Medical men, falling into the deplorable confusion which we have so often pointed out, have applied the remedy right or wrong, by hook or by crook (à tort et à travers), sometimes for one case, sometimes for another, and meeting either with success or failure as chance might happen, and of which they could give no rational account, they have, as might be expected, come to the most opposite conclusions. Thus some practitioners have applied compression to all sorts of cutaneous inflammation alike; to angioleucitis and to phlebitis, to simple erysiplas and to diffused phlegmon; whereas, the remedy, even when judiciously employed, is useful at first only in the treatment of phlegmonous erysipelas, and in some cases of angioleucitis and phlebitis. It is utterly inefficacious in arresting the progress of simple erysipelas."

It is not to be supposed that we quite assent to the truth of these remarks of M. *Velpeau;* compression has always seemed to us a very questionable remedy in any form of cutaneous inflammation, except when the disease is in a chronic or very subacute stage. It is but too true, that we can very rarely allow ourselves to be guided by the French writers as to the *treatment* of *diseases:* they are excellent morbid anatomists and clever operators, but their *ratio* and *methodus medendi* can seldom be imitated with advantage.

*The Nitrate of Silver.*—Our author is not very favourable to this remedy, which has been so much vaunted by English and American writers:—"These gentlemen seem to me to have acted very confusedly; for it is almost impossible to determine in what form of cutaneous inflammation the remedy has been found chiefly useful. I have used it in upwards of thirty cases; in some, applying the caustic to the affected surface directly, in others around its circumference; occasionally the remedy has seemed to be useful, but more frequently it has utterly failed. Sometimes, indeed, it has appeared to arrest the progress of the inflammation; but in most cases this was merely temporary, and the zone of the cicatrization did not ultimately prevent its extension. I have given a fair trial to the remedy by applying it to one limb that was affected with erysipelas, and treating another limb of the same patient in another manner; and I have found that the former was often the more tardy of cure. On the whole, therefore, I regard the nitrate of silver, 'comme un moyen inutile et à laisser de coté.'"

M. *Velpeau* is not more favourable to the use of the acid nitrate of mercury as a local application to erysipelatous surfaces, although it has been recommended by MM. *Biett* and *Cazenave,* and extensively employed by them at the Hospital St. Louis.

He disapproves also of blisters as a remedy for erysipelas, although they were so highly praised by the late Baron *Dupuytren.* He has no better opinion of spirituous and camphorated lotions; or even of the irrigation from a continued stream of cold water, a remedy which has, of late years, been recommended with such enthusiasm by some practitioners. At first the most marvellous cures were effected by this remedy of remedies; but alas! on further trial of it, in the hands of unprepossessed experimenters, it was found *wanting.* We have always reason to distrust a remedy that is announced to have such extraordinary healing powers. The pool of Bethaisda itself could not work greater wonders than the continued irrigation from a stream of cold water on an erysipelatous surface, according to some reports. But the dream has passed away, and now it is discovered that the remedy is not only inefficacious, but is also, in not a few instances, absolutely prejudicial, exposing the invalids to catarrhs, rheumatism, and *milles autres accidens.*

Then again, the *inunction* of *mercurial* and other ointments, a practice that has been so lauded by many of his fellow countrymen, is reported by M. *Velpeau,* after a pretty extensive experience of its use, to be utterly *destitute of any appreciable advantage.* It possesses no power of arresting the inflammatory action; and at best it serves only to abate somewhat of the heat and sense of burning which generally accompany erysipelatous affections.—*Journal des Connaiss. Medicales.*

*Remarks.*—On the local treatment of erysipelas, as indeed on most topics of practical medicine, French writers are certainly not our best advisers. They are so deficient in a knowledge of general and fundamental principles to guide them, that they seem to be tossed to and fro before they can ever decide on almost any subject appertaining to the management of disease. For example, take the present case. We believe that most British practitioners will agree with us, that topical treatment is of secondary importance only in the cure of erysipelas, unless, indeed, where the disease is obviously the result of a local irritation, such as of leech-bites, &c. The question of chief importance, at least when the disease is severe, and accompanied with constitutional disturbance, is whether we are to adopt an antiphlogistic, or a stimulating and tonic treatment internally. Much mischief has been done by laying down too absolutely and dogmatically a certain line of practice, to be followed in all cases of the disease without distinction. Now this is a very serious practical error. One set of cases will be found to require depletion and a lowering regimen; while another set of cases will be best treated with the administration of opium, quinine, beef-tea, and perhaps also wine or brandy. The wise physician will be regulated by the state of symptoms in each case, as well as by the medical constitution of the season, and the type of the prevailing diseases at the time.

With respect to the local treatment, this also will require to be modified according to the character of the constitutional symptoms, as well as by the condition of the affected parts. Perhaps the safest, as well as the most useful, application in the majority of cases will be found to be a weak spirituous lotion, used *tepid*. There cannot be a doubt that considerable risk is incurred by employing any means likely to repel the cutaneous inflammation suddenly; but this is certainly no sufficient reason to reject all topical remedies, or to be satisfied with such innocuous substances as flour, chalk, carded wool, &c. The composition of the lotion may be advantageously varied according to existing circumstances, by the addition, for example, of varying proportions of the liquor plumbi, liquor ammoniæ acetatis, acidum hydrocyanicum, &c. The addition of an ounce or two of spirit of rosemary to eight or ten ounces of camphor mixture will, for a large proportion of cases, make an exceedingly good application—to be used *tepid*. By covering the parts with a piece of oiled skin, they are kept uniformly moist, without being apt to become too much chilled.

As to M. *Velpeau's* opinion, that a solution of the sulphate of iron has any specific effect on erysipelatous inflammation, we need scarcely say that it is quite fanciful.

A solution of sulphate of zinc or of any mild, astringent, would answer quite as well; and moreover have the advantage of not staining the linen of the patient or of his bed.

What must surprize the reader is, that an experienced surgeon like M. *Velpeau* should have such vague notions of therapeutic principles as to write a lengthened paper for the purpose of announcing to the public the discovery of a *nouveau topique* for the treatment of erysipelas. But such is the character of too many of the French school. Excellent anatomists and pathologists they are; but in practical medicine, or, in other words, in the treatment of diseases, they seem to be woefully deficient.—*Rev.*

---

### HINTS FOR THE ADMINISTRATION OF IODINE.

Dr. *Mojsisovitz*, of Vienna, has contributed an elaborate paper to the Medicinische Jahrbucher, on the administration of iodine and its various preparations, in which he has pointed out certain precautions, which are probably not generally known, and the ignorance of which may account for the unsatisfactory results so often derived from their use.

As the feculent matters decompose the preparations of iodine, we find this substance in the state of ioduret of starch, in the stools of persons who eat bread, potatoes, rice, gruel, and vegetables, while taking the medicine. It is therefore necessary to interdict the use of every sort of food containing fecula to patients to whom iodine is given. It is probably owing to a decomposition of this kind having taken place, that we are to explain the inert effects of those enormous doses of the medicine which have been exhibited by some physicians, as Dr. *Elliotson*, Dr. *Buchanan*, of Glasgow, and Professor *Forget*, of Strasbourg. It is rather a curious circumstance that these gentlemen should not have detected traces of the ioduret of starch in the feculent matters of their patients, because Dr. M. informs us that he has never failed to discover it in the stools of such as eat farinaceous substances, while they were taking the medicine.

According to the experience of our author, the use of saline baths greatly promotes the action of iodine on the system. He has also reason to believe that the activity of its operation is a good deal influenced by the condition of the weather at the time. When the air is clear and dry, it seemed to have most effect, and more especially when there was a tendency to inflammatory complaints; whereas, on the other hand, its action seemed to be almost null during the endemic prevalence of smallpox, puerperal fever, and diarrhœa.

The crises, which iodine has a tendency to provoke, are salivation, and a cutaneous eruption like scarlatina or miliaria; the secretion of the urine is usually the more abundant in proportion as the diet of the patient is kept low and restricted.

Dr. M. prefers the hydriodate of potash, and the proto and deuto-iodurets of mercury, to either pure iodine or to any other of its preparations. He regards the tincture of iodine as one of the very worst formulæ that can be used; it is more likely, he says, to cause a wasting of the testicles or mammæ, hæmoptysis, palpitations of the heart, &c. than any of its salts.

The dose of the hydriodate which he recommends for adults is about fifteen grains dissolved in distilled water, in the course of the day.

If there be any open ulcers, they should be kept wetted with a solution of the hydriodate; but if the local affection be a tumor, then he recommends that it should be well rubbed with an ointment composed of 2 parts of the proto-ioduret of mercury and 24 of lard.

The diseases in which Dr. M. has used iodine with advantage are œzena, ulceration of the tongue, palate, &c., various forms of obstinate cutaneous disease and of secondary syphilis, white-swelling and other maladies of the joints, periostitis, tumefaction of the lymphatic glands, scrofulous induration of the subcutaneous cellular tissue, and many of the other kinds of strumous disease.

---

### On Moral Therapeutics.

M. *Reveillé-Parise* is, we believe, well known in Paris as an accomplished and highly literary physician. He is one of the Secretaries of the Royal Academy, and we have observed that, on more than one occasion, he has been solicited to pronounce the funeral *eloge* over some distinguished son of science.

He has recently published, in one of the French periodicals, an elegant Essay on Moral Therapeutics, or the influence which the mind and passions exercise in the production and cure of various diseases. It is certainly a good subject for a medical theme, affording ample scope alike for the observations of the practical physician and the reflections of the thoughtful scholar. It was the remark of Napoleon that, in war, the moral are to the physical means as three to one—so highly did that consummate general rate the influence of mere mind on the issue of any great military enterprize. Now the same will often be found to hold good in the more peaceful operations of the healing art. It is by studying the mind, the feelings, and passions of his patients with more than usual tenderness and sagacity, that one physician so often outstrips another in the extent and success of practice. We believe that the want of such a study is apt to be a besetting sin of those medical men more especially who have been long occupied with hospital practice. M. *Parise* very pertinently remarks on this subject, that "patients in these institutions are almost quite unknown to the physician and the physician to the patients; when they are once discharged, they are completely forgotten; there is no unbosoming of the heart either attempted on the one side, or encouraged on the other. The patient suffers, or is cured, dies or leaves the hospital, bearing within his own breast the arrow that has wounded his feelings, and which has been the cause of the disturbed equilibrium of his bodily functions."

There cannot be a doubt but that psychological causes of disease are too apt to be entirely overlooked in the present day, and that physicians, in their minute examination of all the physical symptoms of a malady, often overlook the influence of mental emotions on its development, its progress, and its termination.

"If a patient dies," says M. P., "we open his body, rummage among the viscera, and scrutinize most narrowly all the organs and tissues, in the hope of discovering lesions of some one sort or another; there is not a small vessel, membrane, cavity, or follicle which is not attentively examined; the colour, the weight, the thickness, the volume, the alteration, nothing escapes the eyes of the studious anatomist. He handles, touches, smells, and looks at everything; then he draws his conclusions one way or the other. One thing only escapes his attention; this is, that he is looking at merely organic effects, forgetting all the while that he must mount higher up to discover their causes. These organic alterations are observed, perhaps, in the body of a person who has suffered deeply from mental distress and anxiety; these have been the energetic cause of his decay; but they cannot be studied in the laboratory or in the amphitheatre."

.... "Many physicians of extensive experience are destitute of the ability of searching out and understanding the moral causes of disease; they cannot read the book of the heart; and yet it is in this book that are inscribed, day by day and hour by hour, all the griefs, and all the miseries, and all the vanities, and all the fears, and all the joys, and all the hopes of man, and in which will be found the most active and incessant principle of that frightful series of organic changes which constitute pathology."

This is quite true; whenever the equilibrium of our moral nature is long or very seriously disturbed, we may rest assured that that of the animal functions will suffer.

Many a disease is the *contre-coup*, so to speak, of a strong moral emotion; the mischief may not be apparent at the time, but its germ will be nevertheless inevitably laid.

"An aneurism of the heart, an engorgement of the liver, a scirrhus of the pylorus, an effusion on the brain, or a softening of some point of its substance, typhoid fever, and the majority of what are called nervous diseases—proceed more or less directly from some, perhaps forgotten, grief, but which, *velut spina in corde,* to use the words of Hippocrates, has gradually destroyed the springs of the living economy. Can we doubt but that it was concentrated chagrin that was the vulture which preyed upon the vitals of Napoleon on the rock of St. Helena?"

It is, indeed, often very difficult to trace distinctly the relation between the cause and the effect, except, perhaps, in our own individual cases, or in those of our immediate friends. But where is the medical man that could not tell many a story of the workings of the mind in unhinging the machinery of the body? His own personal experience probably might furnish him with many such a lesson.

And perhaps our author does not much exaggerate the influence of mental causes when he says, that "deep and protracted distress of mind is the *point de depart* of the greater number of organic diseases.* The learned *Mœringhen* has very justly observed: vix ullus reperitur morbus, cui non aliquod animi pathema, vel ansam, vel incrementum, vel remedium dederit—an axiom as true as it is pregnant with practical results."

1. The organ which is most apt to be affected by mental and moral causes is assuredly the *brain.* We cannot, indeed, point out the connection between the mind and the body, nor explain how they should act and re-act on each other; yet the fact is obvious, every hour that we live. It may puzzle, indeed, the philosopher to understand how it happens that an idea, an entity that is altogether metaphysical, invisible, intangible, without extension or form, or weight, should

* *John Hunter* attributed the disease of the heart, of which he ultimately died after many years' suffering, to the fear of having caught the hydrophobic virus, while dissecting the body of a patient who had died of rabies.

nevertheless act with such force on the body as to prostrate and destroy the stoutest frame. Yet so it is. Take the case of a man who learns that 2000 miles off a vessel which holds all his fortune is wrecked, or that an only child has died: nothing touches him, nothing directly affects his body, but the iron has entered his soul; and soon, nay almost immediately, are the effects of the mental anguish visible in his outward constitution. He experiences a violent, electric-like commotion that shakes every organ of his body; he trembles all over, and feels dizzy in his head; a fixed, deep-seated pain is felt there, that robs him of sleep and chases all his appetite away; and if nothing is done to relieve him, inflammation of the membranes of the brain, or encephaloid congestion, or apoplexy, or palsy, or softening of the brain, or some other fatal mischief, is more or less quickly induced.

2. The *stomach* is the organ, after the brain, most apt to suffer from mental disquietude. The first effect of a deep grief, or even of a sudden and extreme joy, is to suspend the appetite and interrupt the digestive process. This disturbance of the gastric functions is owing to a lesion of the sensory and contractile powers of the stomach, in consequence of the irregularity of its innervation or supply of nervous influence. There seems to be in some cases even a certain amount of paralysis of the organ induced at the time. However this may be, we certainly observe not unfrequently that serious organic mischief of the stomach is apt to follow protracted grief and anxiety of mind. In other cases there is only an intense neuralgic suffering, well known under the term gastrodynia, and the symptoms of which so often simulate scirrhus of the pylorus.* How often do we observe that the worst cases of this complaint, which may have resisted all medical treatment, vanish in a day by the intelligence of some pleasing news, or by having recourse to agreeable change of scene. If not relieved, the atony of the stomach will in almost every case, more or less speedily, be followed by the development of organic mischief somewhere. In one case, it may be the lungs that suffer and phthisis is induced; in another the foundation of cardiac disease is laid; in a third the brain, and in a fourth the liver or mesenteric glands are affected. Sometimes the patient dies from complete marasmus, and yet on dissection no morbid lesion of any organ is discoverable. Such was the case with Madame *Nourrit*, the widow of the celebrated singer at Paris: an inward grief followed the melancholy death of her husband, and gradually exhausted all the vital energies of the system, and she died of marasmus.

3. "The *intestines*," says M. Parise, "seem to be less affected by mental and moral influences than the stomach. May this be owing to their being farther removed from the gastric plexus?"

There are however, it must be confessed, not a few intestinal disorders which are very intimately connected with the depressing emotions of the mind. Not to dwell upon various affections of the liver, congestions of the spleen, constipation or irregular action of the bowels, we may allude more particularly to the common disease of *piles*. How very generally is this troublesome complaint associated with an irritable, unhappy, and peevish state of mind? We have often predicted the existence of hæmorrhoids in patients, whom we had never seen before, from merely observing the anxious expression of their countenance.

4. Although the *heart* is not in a physiological point of view now regarded as

* One form of this most distressing malady is not unfrequent in medical and other students who are preparing for their examinations, and who allow their minds to be over-anxious about the result. As a general remark, excessive application of the mind, especially when coupled with anxiety or grief, inevitably deranges the digestive functions.—*Rev.*

the seat of the passions, as the language of poets and moralists would imply, no one can deny that it is powerfully influenced by them. Authors have differed as to how far this influence is more or less direct and immediate on the centre of the circulation. Every one knows that any violent emotion of the mind, whether this be of an exciting or of a depressing nature, is accompanied with a feeling of oppression and distress in the cardiac region, and with a greater or less degree of tumultuous or irregular action of the heart.

"Although the nerves of the heart are by no means numerous or large, it is nevertheless certain that, under the influence of any strong emotion, their action is almost immediately disturbed.* It is therefore readily conceivable that a frequent repetition of such disturbance will, in course of time, be followed by the induction of a permanent disease of the organ. There are perhaps few cases of aneurism of the heart which are not attributable to moral causes; and when we use the common expression that intense grief is *a heart-break,* the phrase is often true in a physical as well as in a moral point of view. The celebrated chemist, *Fourcroy,* is a striking example of the truth of this remark. *Napoleon* had long promised him the situation of rector of the university, but at length he bestowed it on *Fontanes. Fourcroy* was so deeply affected by this conduct, that all the symptoms of a heart complaint became immediately aggravated; and, while in the act of signing some public papers, he cried out *je suis mort,* and immediately expired in the arms of an attendant."

We may here observe that one of the corporeal or physical signs of prolonged distress of mind is a marked predominance of the venous over the arterial system. This is doubtless owing to a diminution of the contractility of the heart, and to the consequent *stasis* or languor of the general circulation. The French proverb—*qui voit ses veines, voit ses peines*—is therefore strictly true.

The influence of the mental emotions on the functions of the *kidney* is strikingly exemplified in almost every case of hysteria. It is not indeed easy to explain why there should be such a copious secretion of limpid urine at the decline of an hysterical paroxysm; but that such is the case, every tyro in medicine knows.†

The *mamma* is another organ, whose secretion is very decidedly affected by the state of the mind and feelings.

"The milk," says M. *Parise,* "becomes suddenly fluid and aqueous, or thickened and acid, or diminished in quantity, or even totally suppressed, returning perhaps as quickly again, by various mental causes, without the slightest appreciable change in the mammary glands themselves. These phenomena we

---

* Few writers on the physiology or pathology of the heart seem to us to pay sufficient attention to the intimate connexion of its functions with the state of the respiration; and yet every disturbance of the breathing is necessarily followed by some irregularity in the cardiac circulation. After a good deal of reflection, it appears to us that the influence of moral impressions on the heart can scarcely be regarded as direct and immediate, at least in the majority of instances; but rather that this influence is exerted primarily on the lungs, and only consecutively on the heart. Attend, for example, to the influence of fear: the breathing is immediately suspended, the throbbings of the heart follow. It is to Mr. *Wardrop* that the profession is especially indebted for the most lucid exposition of this and such-like phenomena: vide his ingenious work on the heart.—*Rev.*

† We have seen more than one case where diabetes mellitus *seemed* to have been brought on by protracted anxiety and distress of mind. In these cases the spinal marrow appeared to be a good deal at fault, as there was a very marked debility of the lower extremities present at the same time. The cases did well under the influence of animal diet and of change of scene.—*Rev.*

observe almost daily in women who are of a highly nervous and impressionable temperament. An excessive, but ill-balanced, tenderness on the part of a nurse often unfits her from suckling her child with advantage. If *Rousseau* had been acquainted with the laws of the animal economy, he would not have insisted, so urgently as he has done, that every mother, without exception, should suckle her children: his eloquent appeal has on many an occasion proved most hurtful to both."

The *cutaneous* functions are very notably affected by the emotions of the mind. How chilly becomes the skin, when grief is preying on the heart! and, on the contrary, how the whole surface glows with warmth when the soul is filled with joy! Captain *Ross*, in the narrative of his arctic voyage, alludes particularly to the circumstance of mental depression rendering the body more susceptible to the impression of cold, and mentions that several of his men became morbidly irritable: the accounts of the disastrous retreat of the French from Moscow, and of the awful shipwreck of the *Medusa* frigate, afford still more striking instances of the same state.

If our space permitted, it would be easy to extend these observations; but as our object at present is not to write a dissertation, but only to offer a few suggestions to our readers, it is unnecessary to say more than urgently to impress on all medical men the necessity of studying the psychological causes of disease. —*Bulletin de Therapeutique.*

### Auscultatory Test for the Administration of Iron.

Dr. *Carriere*, one of the professors of the medical school at Strasbourg, announces as the result of an extensive clinical experience, that in almost every case in which a ferruginous preparation has been found distinctly useful for the relief of nervous disorders, and more especially the Protean class of the neuralgiæ, "one single symptom has been found to be constant and uniform, and has always served as a touch-stone for the treatment to be employed, viz. the blowing-sound or *bruit du diable* in the large arterial trunks." "This precious sign," he continues, "has on many embarrassing occasions sufficed to establish a correct diagnosis of a case, and to lay the basis of a treatment which, although seemingly at the time empirical, has been followed with remakable sućcess. I had often observed the presence of gastralgia and other forms of neuralgic distress complicating the chlorotic condition, and that ferruginous medicines, if properly administered, generally relieved both one and the other affection. I concluded that these concomitant maladies were under the influence of that peculiar alteration of the blood which, in my opinion, constitutes the chlorotic state; and, as in this morbid state I have almost uniformly been able to detect the existence of the *bruit du diable* in the large arteries, I was led to assimilate with it the majority, at least, of the affections that happen to be accompanied with this auscultatory phenomenon, and consequently to employ the same remedies for the relief of all. Experience has justified the correctness of this reasoning. I have successfully treated with steel an immense number of most obstinate neuralgiæ, including many cases of most severe cephalalgia, which had continued for months and even years to resist every other mode of treatment; and I must acknowledge that the presence of the arterial *bruit* has often been the only existing symptom to guide me in my diagnosis, when all the usual symptoms of chlorosis—as, for example, the pallor of the complexion, the absence or deficiency of the catamenia, &c.—were awanting."

Dr. *Carriere* is quite correct in asserting, that many of the most troublesome symptoms which usually accompany genuine chlorosis are often observed in patients, in whom there is no irregularity of the uterine functions, and who do not exhibit the well-known waxy hue of what is popularly called green sickness.

Of these concomitant symptoms perhaps none are so unmanageable as the various forms of neuralgiæ; and the profession would certainly be much indebted to any one who could point out with accuracy a successful mode of treating them, or even furnish them with a means of distinguishing those cases in which we may promise a speedy cure from those which will probably be more unyielding. This diagnostic test or touchstone may be found, in our author's opinion, in the presence or absence of the arterial *bruit;* and he strives to demonstrate that it is by the presence or absence of this auscultatory phenomenon that the physician is to be guided in the selection of his remedies. When once he detects the arterial *bruit* or murmur, he may be satisfied, says Dr. *Carriere*, that steel is the proper remedy for the existing disease, and that the use of it is required as long as the bruit can be heard. The following case is given in illustration.

A young girl, who had never menstruated, and who was of a delicate nervous constitution, was attacked with catalepsy, the paroxysms of which usually returned every third or fourth day. As I found a distinct *ronflement* in the carotid and subclavian arteries, I at once prescribed the use of steel remedies, at first in small doses, to be gradually increased in quantity. The attacks gradually became less frequent and less severe, and I uniformly found that the arterial murmur diminished in a corresponding degree. The use of the steel having been, contrary to my advice, suspended for some time, the paroxysms returned with greater frequency, and the *bruit du diable* recovered its former intensity.

The same circumstance was repeated three or four times subsequently, and the coincidence between the aggravation of the malady and of the arterial sound was very remarkable on every occasion, without exception. The cure is still not complete, although the disease is much mitigated; for the auscultatory phenomena continues, although with considerably less intensity. I feel confident that if the use of the steel was persevered in, the disease might be altogether subdued.

Here is another and a still more satisfactory case; it was one of *hemicrania.*

A lady, 23 years of age, had been subject from her fourteenth or fifteenth year to attacks of excessively severe hemicrania, returning at irregular intervals of time, and seemingly connected more or less with atmospheric variations. During the attacks, the patient was obliged to keep her bed, in consequence of the severity of the pain. As may be supposed, a great variety of remedial means had been tried, but hitherto with little decided success. On my first visit, I directed my attention to ascertain if any abnormal *bruit* could be detected in the subclavian or carotid arteries. At once I detected the characteristic sound, and thence determined, without delay, to administer steel. It should be stated that there was no irregularity of the catamenial function, nor any outward sign of chlorotic disease. The use of the carbonate of iron was continued for upwards of five months, at which period all tendency to return of the headaches seemed to be completely removed. The gradual subsidence of the arterial *bruit* was very remarkable during the course of the treatment.

In another case, in which the disease was an intercostal neuralgia of nearly four years' standing, the same mode of treatment was adopted, and with similar success, after all other remedial means had failed.

Perhaps the most valuable part of M. *Carriere's* paper is that which treats of that very insidious and often very intractable class of complaints, well known by the name of *gastralgiæ.* He says:

"The history of these most troublesome affections is certainly one of the points of pathology that is very imperfectly understood. Their nature and ætiology are still very obscure, the rationale of their symptoms is vague and imperfect, and their appropriate treatment is most uncertain and unsatisfactory.

A number of morbid affections, very different the one from the other in their nature and their origin, are classed together, as if they were degrees only of the same morbid state. When the physician determines that it is not a case of gastritis, he at once pronounces it to be a *gastralgia,* and there his diagnosis ends. As might be anticipated, the treatment is apt to be very empirical."......

"The essential point is to know how to discriminate the cases in which we may reasonably anticipate benefit from the use of iron. Now here, experience comes to shew that this very valuable remedy is found to succeed well only in those gastralgiæ, which are connected with a chlorotic condition of the system, whether the usual symptoms of chlorosis be present, or not, provided the characteristic *bruit du diable* can be detected in the large arteries.* Besides, this form of gastralgia has often a peculiar and characteristic physiognomy, by which it may be distinguished from the varieties of the disease. Its principal features are the continuity of its symptoms, or its alternations with some other neuralgia; the great sensibility of the epigastrium, the difficulty or impossibility of digesting food, even of the lightest kind, a sense of weight in the region of the stomach, the frequent and inodorous eructations after eating, &c.; to these symptoms is sometimes added a neuralgia of the supra-orbital nerve, or perhaps a genuine hemicrania."

Dr. *Carriere,* in alluding to the mistake frequently made by some of his countrymen, of confounding gastralgia with gastritis, remarks that "the latter affection is as rare as the former one is frequent." He is perfectly correct in this statement; we believe that inflammatory affections of the stomach are of very rare occurrence indeed, and may generally be discriminated with ease by the judicious physician: there is always more or less of feverish irritation, indicated by the state of the pulse, the condition of the urine, the thirst, &c. attending it; whereas, these symptoms are rarely present in genuine uncomplicated gastralgia. Dr. C.'s exposure of the fallacy of the Broussai-ists is so graphic that we are tempted to give it at length:—

"There are still in the present day not a few physicians who, regarding pathology through the prism of the physiological doctrine, are ever following after gastritis and gastro-enteritis—those two phantoms which, like genuine Protei, are shewing themselves under a variety of forms, and against which they fancy that they are continually waging warfare. It is not difficult to foresee what will happen, when these gentlemen have to do with the class of complaints which we are at present considering. The sensitiveness of the epigastric region, the uneasiness felt during digestion, the increase of pain after eating even the lightest and blandest food, the excitement and erethism of the nervous system which not unfrequently give rise to a quickened and even a hard state of the pulse—oh! these symptoms must necessarily indicate the existence of *gastrite;* and forthwith leeches, and poultices, and mucilaginous drinks and low diet must immediately be had recourse to. But the complaint does not yield; it rather becomes worse: the weakness is greater, and the catamenia, which may have been regular hitherto, are suppressed. These changes are attributed to some imprudence on the part of the patient—leeches are again ordered, and the starving regimen is once more enjoined. In this way, a patient, who under proper treatment might have recovered her health in a week or two, is reduced to a state of such debility and bloodlessness that it will require months before she can be

* It will be observed that Dr. *Carriere* does not use the term *chlorosis* in its ordinary acceptation. He employs it to designate a state of system which he considers to be connected with a certain special state of the blood, but which is not necessarily indicated by a waxy pallor of the countenance, or by any irregularity of the menses. This morbid state, although much more common in the female than in the male sex, is not unfrequently observed in the latter.

well. It is in such often-mistaken cases as these, that the judicious exhibition of ferruginous remedies often acts with marvellous benefit."

Dr. *Carriere* then remarks on some of the cases which he has met with in the male sex, as requiring the use of steel. One was in a young man who had recently been treated at La Charité Hospital* for typhoid fever, with repeated venesection and other debilitating measures. Two other patients, of 17 and 21 years of age, had suffered repeatedly from attacks of ague, from having resided for a length of time in a marshy unwholesome district. A fourth was in a state of great debility, induced by a protracted diarrhœa; and a fifth had very seriously injured his health by having given way to the abominable practice of masturbation. Of these *five* patients, *two* suffered from most severe cephalalgia, and *two* from gastralgia: in *all* of them, there was great muscular debility; in all, a blowing sound might be distinctly perceived in the subclavian and carotid arteries; and all were speedily cured by the use of ferruginous remedies.

Who can therefore doubt that there is a very intimate analogy between such cases in the male sex, and the much more frequent ones in the female sex, which are so generally classed under the term either of chlorotic or of hysterical disease?

"In conclusion," says our author, "it is in my opinion abundantly demonstrated, that certain gastralgiæ, certain neuralgiæ, and several forms of neurosis, which are not unfrequent in young persons of either sex, and which are accompanied with a *bruit du diable* in the large arteries, are so many phenomena which should be considered as the pathological expression of a *dyscrasis* of the blood, which essentially constitutes the disease of chlorosis, such as it is understood in the present day.

These various pathological expressions of the morbid condition of the blood in question may almost uniformly be removed by the judicious administration of ferruginous remedies, continued for a sufficient length of time until the system is saturated, or, in other words, until the auscultatory phenomenon, alluded to, has completely disappeared."

Dr. *Carriere* admits that he has occasionally detected the presence of the arterial *bruit* in cases, which he should not think of attributing to the same condition of the blood as that he has been treating of. For example, he has found it in two cases of dropsy induced by diseased liver: in one case of *Bright's* disease of the kidneys; and in one case also of diabetes mellitus. Moreover it is not unfrequent during the early period of pregnancy.—*Bulletin de Therapeutique.*

We cannot answer for the correctness of Dr. *Carriere's* views in reference to the arterial *bruit* being so good a semeiological test for the administration of steel medicines as he alleges it to be. We should think the idea to be more ingenious than true. Certain however it is, that steel is by far the best remedy in such cases as those alluded to by our author; and in this respect our experience entirely coincides with the results of his. Many very unmanageable cases of gastralgia and of various forms of nervous suffering will yield to it, after having resisted all other sorts of medicines.—*Rev.*

---

* We may remind our readers that this is M. Bouillaud's hospital, and the head quarters therefore of the new *formula* of bleeding *coup sur coup*—a discovery, the value of which the animated doctor no doubt thinks will rival that of vaccination.—*Rev.*

### Treatment of Phthisis with Arsenical Cigarettos.

M. *Trousseau* is the author of the new proposal for treating pulmonary consumption with smoking cigars which have been impregnated with a minute proportion of arsenic. The worthy professor commences his observations with remarking, that however serious and unyielding any disease may have hitherto proved, we should never think of abandoning it to its own course; for it may so happen that, in the trial of various plans, some one thing or another may be found out, if not to cure, at least to relieve, the evil. "If," says he, "we have been faithful in our own practice to this principle, we have been especially so in reference to tuberculous affections of the lungs. We have tried, and then in its turn abandoned, the use of the extract of *cachou* in considerable doses, the rhatany; almost every form of tonic medicines; caustic issues, and various other kinds of drains over the seat of the disease; the sulphuret of calcium, the medicinal waters of Enghien, of Bonne, &c.—but all these and many others, without arresting the progress of the disease."

What a medley of incongruous remedies, and all for the same complaint! Well may such practice suggest the idea of the random strokes of a man to hit his enemy in the dark. Really such empirical practice is unworthy almost of an advertising quack: what then shall we say of an established professor coolly announcing it as the result of experience! The first suggestion of the arsenical cigars occurred to his mind in the following way:—

"A physician from one of the departments called upon me to say that he had discovered a method of curing pulmonary consumption, and to request me to intrust to his care some patients to try the effects of his medication under my inspection—adding, however, that he meaned to make *une affaire* of his discovery. I could not accede to such a proposition, unless upon the distinct understanding that every thing should be made public. At first he assented to my wishes but afterwards drew back, confessing, however, that his remedies consisted chiefly in irritating fumigations or inhalations. Acting upon this hint, I have tried a variety of means in this way, and among others, little cigars impregnated with a minute proportion of arsenic."

He goes on to remark:—

"We have no doubt as to the curability of tubercles of the lungs. The post-mortem examination of many old subjects, exhibits most distinct traces of cicatrised caverns. As there is a great analogy between scrofula and pulmonary tubercles, we at first directed our attention chiefly to those substances which are known to be useful in promoting the healing of scrofulous ulcers. You know that if a small quantity of the Vienna powder be applied upon the surface, the skin there becomes disorganised, and that below the eschar we observe a cicatrix recently formed. But if we use a less powerful caustic, like the arsenical or chloride of antimony pommade, we may succeed equally well in effecting the cure of a scrofulous ulcer.

Then again, of all remedial means against cancer of the skin, certainly none on the whole is so trustworthy as arsenic: it seems to act in two ways; 1, as a caustic, and 2, as an alterative of the existing morbid process. Reasoning on these facts, I was led to conclude that this most potent remedy might be used with advantage in phthisis pulmonalis; and I was confirmed in this idea by finding that many of the old writers on medicine have mentioned the curative effects of certain remedies, which are known to contain some preparation of arsenic in cases of wasting of the lungs."

M. *Trousseau* thus describes the method of employing arsenical fumigation, which he at first adopted:

"I dissolved one or two *grammes*—a *gramme* is equivalent to nearly 15½ grains—of the arseniate of potash in 10 grammes of water; two grammes of the solution were then poured upon a sheet of paper, and when this was well wetted, it was allowed to dry. Of this prepared paper a number of small *cigarettos* were made. The patient is advised to draw in a large mouthful of the smoke, and inhale it as deeply as he can, so as to cause it to penetrate into the bronchi. The effort causes a good deal of coughing at first, but becomes gradually more and more easy. The inhalation is repeated two, three, four, or five times, according as the patient can bear it."

M. *Trousseau* administers at the same time minute doses of arsenic internally, and strongly recommends the simultaneous use of hemlock plasters—the extract spread upon diachylon plaster—on the chest. He narrates two or three cases, in which the use of these means appeared to exert the most pleasing effects in mitigating the symptoms, and in decidedly arresting the progress of phthisis in its advanced and hectic stage.

"We do not pretend," concludes our author, "to cure tubercular disease of the lungs with arsenic; all that we maintain is, that it is by means of it that we are enabled to modify the constitutional symptoms, and greatly improve the general health of the patient, so that life may not only be rendered more comfortable, but may be considerably prolonged."—*Gazette des Hôpitaux.*

*Remark.*—The practice of inhalation in the treatment of phthisis pulmonalis is perhaps too much neglected on the whole. The extravagant encomiums which some writers have passed upon inhaling the vapours of iodine, chlorine, &c. have no doubt contributed to this end. Without anticipating too much from the ingenious suggestion of M. *Trousseau,* we really do think that it deserves a cautious trial in conjunction, as a matter of course, with other local means, and those constitutional remedies that are calculated to give a healthy tone to the system. Arsenic is certainly one of the most powerfully alterative tonics which we know.—*Rev.*

---

### REMARKS ON VARIOUS REMEDIES IN PHTHISIS.

We extract the following very sensible observations from a paper on the treatment of incipient consumption in the January Number of the Gazette Medicale de Paris.

*Emetics* are certainly among the most useful of all remedies in the earlier stages of the disease. The less advanced that the disease is, and the more *chronic* it is in its character, the more benefit may be expected from a judicious administration of emetics. Many a case, in which there could be little doubt but that tubercular disease of the lungs was developing itself, has done well under this practice, in conjunction with the use of other appropriate means, and all the symptoms of the impending mischief have been arrested; even in old chronic cases, where there was a marked dulness on percussion under one or both clavicles, but where there was no sign of softened tubercles or of vomicæ, the most pleasing results have not unfrequently been obtained. On the contrary, where the disease is acute, or accompanied with feverish irritation, emetics have not produced the same amount of benefit; and no permanent advantage can be expected from their use, when the hectic fever is fairly established, and the strength of the patient is much reduced. The emetic should be taken by the patient early in the morning, before breakfast, and while he is still in bed, so that he may have an hour or two's repose after the fatigue of the vomiting. It may be repeated every morning, or only every second or third one or so, for several weeks. Generally the patient feels more comfortable on those days on which he has been vomited, and the cough and other symptoms are decidedly mitigated.

A full dose of ipecacuan, either alone or combined with a few grains of sulphate of zinc, is as good a formula as we can employ.

*On the whole, emetics are certainly the most eminently efficacious remedies which I have seen employed in the treatment of incipient phthisis, and, in conjunction with other means, they seem to hold out the promise of, if not curing, certainly arresting the progress of the disease.*

*Sanguineous Evacuations.*—In phthisis, unaccompanied with an inflammatory affection of the lungs or of the pleura, or with hæmoptysis, we should certainly avoid any depletions of blood. Much mischief has been done, both in former times as well as more recently, by having recourse to cuppings, leechings, and even bleeding from the arm, in almost all cases of pulmonary tubercle, as if the disease was of a genuine inflammatory nature.

Even when we are obliged to resort to sanguineous depletion, in consequence of the complication of inflammation or of hæmorrhage, we should be content with obtaining a few ounces from the upper part of the chest by means of leeches or of the cupping-glasses. Minute doses of the tartrate of antimony, combined either with the syrup of poppies or with the extract of hemlock or of henbane, should at the same time be administered for a few days, until the febrile symptoms abate and the cough becomes looser and easier.

*Counter-irritants.*—Much relief may often be derived from the use of these means;—from blisters when the symptoms are rather acute, and from the antimonial ointment in more chronic cases. The irritant recommended by Dr. *Stokes*, which is composed of acetic acid and essence of turpentine, is a most useful one, and may be often most advantageously substituted for those in more common use: it seems indeed to have some other effect than that merely of a counter-irritant.

*Iodine.*—Decided benefit has been obtained in not a few cases of tuberculous disease of the lungs, as well as in various other allied forms of scrofula, from the use of this potent remedy and of its preparations. Minute doses of the iodine itself, in combination with the hydriodate of potash, may be given in distilled water or in the infusion of Calumba, to which some syrup of poppies should be added. The medicine should be continued for a length of time, as its action is slow and progressive.

*Tonics* are necessary in almost every case of phthisis, where the disease is not acute or febrile. Steel, quinine, nourishing diet, country air, &c. are the best that can be used.

It is unnecessary to say that the exhibition of this and of every other class of medicines in phthisis requires great tact and practical experience. No one but an empiric can talk of any remedy being a cure for consumption. The following judicious remarks shew that the author is a man of judgment: there is nothing novel in them, but there is, what is much better, good sound practical sense.

"When a person, who may have a hereditary predisposition to tuberculous disease, is suffering from a troublesome cough, accompanied with expectoration, dyspnœa and feverish restlessness, the first thing that we should do is to order the application of a few leeches immediately above or below the clavicles, and small doses of the tartrate of antimony with henbane or hemlock in frequently repeated doses: a saline purgative may also be necessary. If the symptoms do not abate, we must again have recourse to the leeching, or to a small blister. When the symptoms of bronchitis have ceased, as usually happens in the course of a few days, but yet a hard dry cough remains, and the inspiratory murmur be rough and noisy, or that of expiration be louder and more prolonged than in health, the use of an emetic every second morning before breakfast will be found to be of the greatest service: the preparations of iodine internally, and the turpentine and acetic acid liniment, may also be used with advantage, at the same time. After this treatment has been continued for some weeks, the patient will usually be found to be much relieved of all his constitutional distress; and perhaps a pallor of the countenance and a quickened state of the pulse are the chief, if not the only, symptoms that are present. Now is the proper time for the exhibition of tonics, and the use of a nutritious diet: residence in the country at the same time should be had recourse to, if possible. Under such a course of treatment the patient will often regain his strength and flesh, and perhaps all the auscultatory signs of pulmonary tubercles will cease, except a certain hardness or roughness of the respiratory sounds.

Even in the hæmorrhagic form of phthisis, the use of occasional emetics has been attended with decided benefit, when the bleeding has ceased: the efforts of vomiting do not seem to have any tendency to reproduce the hæmorrhage."—*Gazette Medicale.*

---

### M. TROUSSEAU ON HOOPING-COUGH.

The following remarks, though desultory and unconnected, are nevertheless worthy of notice.

"Pertussis is occasionally ushered in with an almost incessant tickling cough, which leaves the patient scarcely five minutes' repose at a time. We were lately sent for by a lady, residing in the Chaussée d'Antin, who had been seized with feverishness, pain in the throat, and a cough so incessant that she had not three minutes' respite. After a few days' suffering in this way, the cough assumed a hooping character, and then there could not be any mistake as to the nature of the disease. At the same time we had under our care a young child, who was affected in a similar manner for ten or twelve days, before the regular hooping-cough was developed. It is useful therefore to keep in mind that, during some epidemics, the disease is thus ushered in; otherwise a medical man might be a good deal puzzled what to make of such a train of symptoms."

*The Severity of the Disease not proportioned to the Bronchitic Disturbance.*

"A number of physicians have supposed that the accumulation of mucosities in the bronchi was the cause of the fits of hooping-cough, and that the severity of these was always proportionate to the quantity of the secretion. Auscultation disproves the correctness of this idea; for very often, when the fits of coughing are both frequent and very severe, we can perceive only an indistinct and feeble mucous *rale.* The larger proportion of the sputa rejected during a fit of hooping-cough certainly does not proceed from the bronchi.

Again, if pertussis is to be considered, as the physiological school pretends, as a species of catarrh accompanied with convulsive paroxysms of coughing, it would surely be observed that the nervous symptoms increased in severity with the increase of the catarrhal symptoms. The physiological physicians have said, that in follicular enteritis the cerebral phenomena are owing to the sympathetic *retentissement* of the intestinal irritation on the encephalon. In some circumstances this may be so; but as a general position we cannot well admit that the gravity of the two sets of symptoms is at all proportionate to each other."

. . . . . . "In hooping-cough, it is generally observed that its characteristic phenomena become less distinct just in proportion as the constitutional disturbance is greater, and the pneumonic or bronchitic symptoms are more severe. Whenever a patient is in the condition of plethora, the tendency to the development of nervous disease becomes less: spasmodic symptoms are almost always observed to diminish as the tendency to fever is increased."

. . . . . . . "Whenever you find the hooping in a case of pertussis rapidly subside and the feverish symptoms increase, there is reason to apprehend an attack of some phlegmasia. The disease, it should be remembered, is essentially of long duration, and *on ne rompt qu'en faisant toujours face.* As the inflammatory symptoms subside, the hooping character of the cough usually returns more severely than before."

Alluding to the case of a child, in whom pneumonia supervened while it was affected with pertussis, M. *Trousseau* remarks:

"Now, in ausculting our young patient, we find at the root of the left lung behind a sub-crepitant rale, and on the right side a subcrepitant and mucous *rale.* This infant has lobular pneumonia. .... The sub-crepitant rale is an indication of pulmonary catarrh, while the crepitant rale indicates pneumonia; the former is perceptible both during inspiration and expiration, but the latter is to be heard only during inspiration. In our patient we hear the subcrepitant rale over almost the entire extent of the chest, whereas the crepitant rale exists only at some points: we therefore infer the existence of lobular pneumonia."

*Curative Influence of Emetics.*

"To most of the children we have given one or more vomits of ipecacuan, and in all with the effect of decidedly abridging both the frequency and the severity of the coughing fits. The vomit should be repeated every second day in most cases. We usually administer at the same time repeated doses of a mixture of equal parts of the syrups of valerian, belladonna, and opium, with a portion of æther added to it. Do not however expect to cure the disease either with this or with any other mode of treatment; all that we can hope to do is to diminish its severity."

. . . . . . "We find it recorded by several authors that vaccination usually mitigates very decidedly the severity of the paroxysms of hooping-cough. In some cases, where it has been practised soon after the appearance of the disease, this has very quickly subsided. If such be the case with vaccination, we may reasonably expect a similar result from the supervention of small-pox; and such appeared certainly to be the case in one of our patients. Such a morbid eruption is a much more efficacious revulsive than any artificial one, such as that produced by the ointment of the tartrate of antimony."—*Gazette des Hôpitaux.*

---

### Clinical Observations on Rheumatism. By M. Chomel.

The following statistical results are derived from the clinical wards at the Hôtel Dieu, under the care of M. *Chomel.* During the sessional (scolaire) year 1840-41.

During the course of the year, 31 cases of acute articular rheumatism were admitted: of these 17 occurred in men and 14 in women. One half of the cases occurred during the Summer, and the other half during the Winter months. In the majority of the cases it was extremely difficult to determine the exciting cause of the disease: seven or eight only of the patients attributed it to the influence of cold. The medium duration of the disease was about 20 days: it seemed to be rather less in Winter than in Summer.

Of the 31 cases, there was one only in which the patient had exhibited symptoms of cardiac disease (palpitations and attacks of dyspnœa) before the invasion of the rheumatic attack. In another patient, immediately after the attack, symptoms of cardiac disturbance developed themselves.

From our experience, says M. Chomel, during the last four years, during which time 86 rheumatic patients have been admitted into our wards, it follows that six out of this number had been affected with heart-complaints before the attack upon the joints; and that four only have had organic affections subsequently to their first suffering with rheumatism.*

It would therefore appear that the proportion of patients, in whom organic disease of the heart supervenes after acute rheumatism, is very much smaller than that indicated by M. *Bouillaud* and many other writers.

It follows also from our observations that inflammation of the investing membrane of the heart, whether of the pericardium or of the endocardium, is far from being a constant accompaniment of acute rheumatism; for, if it were so, surely organic diseases of the heart should be much more than they really are. The knowledge of this fact ought to guard the minds of medical men against the too positive assertions that have been laid down in some recent works.

*Bruit de Souffle.*—In seven only of the 31 patients was this blowing sound to be perceived; and out of the entire number of our patients since 1837, viz. 86, it was perceptible in 29 only of the cases—or in about one in every three cases.

But to ascertain still more exactly the value of this auscultatory phenomenon, and to determine how far it is so intimately associated with rheumatism, as has been alleged by many writers, we have endeavoured to find out whether it is not present in other acute diseases, in which there is no reason to suspect any complication of cardiac disturbance.

The following are the results of our observations during the past year. We have met with the *bruit de souffle.*

1. In five cases of pneumonia, three of which recovered and two proved fatal. In one of the fatal cases, a very slight thickening of the sigmoid valves was found on dissection; and in the other, in which the blowing sound had been so strong as to make us suspect the existence of an endocarditis, not the slightest trace of morbid change in any part of the heart could be detected.

2. In three patients affected with small-pox, the *bruit* was distinctly heard: it ceased with the disease, without leaving behind any symptom of cardiac complaint.

3. In one case of fatal typhoid fever; on dissection no trace of lesion in any part of the heart was discoverable.

4. In two cases of bronchitis, and in several cases also of acute metritis, the blowing *bruit* accompanying the first sound of the heart was distinctly perceived.

From these observations we are warranted in the conclusion, not only that this auscultatory phenomenon is far from being constant in acute rheumatism, but also that it is not unfrequently present in other acute diseases.—*Gazette des Hôpitaux.*

---

### TREATMENT OF ACUTE RHEUMATISM WITH LARGE DOSES OF THE NITRATE OF POTASS.

Dr. *Brocklesby* seems to have been the first to point out the curative efficacy of saltpetre in the treatment of acute articular rheumatism. In his economical and

---

* It must certainly be rather difficult to substantiate such an assertion in reference more especially to hospital practice; as it is well known that the patients are almost always lost sight of after their discharge from the institution. How then can we determine with any degree of accuracy that in four instances only out of 86 cases of acute rheumatism did any cardiac disease supervene?

In our opinion, M. *Chomel* is as much inclined to under-estimate as M. *Bouillaud* is to over-estimate the frequency of what the latter author calls the coincidence between acute rheumatism and cardiac disease: *in medio tutissimus ibis.—Rev.*

medical observations, published in 1764, he has explained the practice which he found to he most useful. After taking a free bleeding from the arm, when the patient is young and robust, he orders a very copious allowance of warm gruel, in which from one to two drachms of the salt have been dissolved; the patient is to take large draughts of this drink at short intervals. He has given as much as from six to ten drachms of the nitrate, dissolved in from three to six quarts of the gruel, during the course of 24 hours. This mode of treatment rarely fails in producing a great relief of all the symptoms in two or three days; and very often a complete cure of the disease is effected, without having recourse to any other mode of treatment, within a week from commencing the treatment. The nitred diluent usually causes very profuse perspiration, and generally also acts upon the bowels; if it does not, an aperient enema should be given occasionally.

It is to be observed that Dr. *Brocklesby's* remarks are drawn from practice in military hospitals, where the patients are usually robust and healthy.

In 1772, Dr. *Macbride*, in his introduction to the theory and practice of medicine, recommended the same mode of treatment. Two years later Dr. William White (Observations on the use of Antimonial Preparations) makes the following remarks on the effects of large doses of saltpetre:

"The employment of this salt, after bloodletting, is very useful in the treatment of acute rheumatism; but it must be given to the extent of an ounce in the 24 hours, if the vascular irritation be considerable; small doses are of little service. In many cases of chronic rheumatism also, it is of great efficacy. Administered in the dose of from one ounce to an ounce and a half in 24 hours, it often cures the most severe cases of the disease, which may have resisted every other mode of treatment."

The use of the nitrate, notwithstanding such decided recommendations, fell into neglect, and although noticed by M. *Bosquillon* in his translation of *Cullen*, it was not till 1832 when it was again brought to notice by M. *Gendrin*. One of his *internes* at the Beaujon Hospital, M. *Aran*, has brought together the reports of a dozen of cases treated with the nitrate of potash in full doses, dissolved in copious draughts of a mild demulcent diluent, very nearly according to the directions of Dr. *Brocklesby*. The dose of the salt varied from four to eight or ten drachms in the course of the 24 hours. The average duration of the treatment seems to have been about a week. In many of the cases there were well-marked symptoms indicating disease of the heart: these symptoms usually subsided with the suffering in the joints.

The *modus operandi* of the remedy was usually as a powerful diuretic and diaphoretic: occasionally too it acted on the bowels.—*Journal des Connoissances Medico-Chirurgicales.*

*Remarks.*—We are generally pleased with the *reproduction* of any of the remedies which were much used and recommended by the sound practical physicians of the last century, as most assuredly these gentlemen were much less influenced by pathological theories in their treatment of diseases than most of the writers of modern times. That full doses of the nitrate of potash should act beneficially in acute rheumatism, we are quite prepared to expect: for it will be found that all powerfully evacuant remedies, more especially those which act on the skin and kidneys, are more or less useful in this disease. One of the most generally useful of all remedies, in a large proportion of rheumatic cases, is the mistura guaiaci of the London Pharmacopœia; and it is observed that it is usually most beneficial when it acts on the bowels and kidneys. The vinum colchici, or the Dover's powder, may often be added with much advantage to it. Moderate bloodletting, and the exhibition of calomel at bed time, with or without opium, should seldom be neglected at the same time.—*Rev.*

### On the Treatment of Worms.

1. *Ascarides.*—Aperient medicines, although they are not to be trusted to alone for the removal of these worms, are generally necessary. Strong drastic purgatives should be avoided. Rhubarb and aloes, with the addition of a small portion of some mercurial, may be given over night, and in the morning a draught of Epsom salts in a bitter infusion will be found to add to their efficacy. Instead of administering much medicine by the mouth, it is better to use enemata frequently. Common table salt, the muriate of soda, dissolved in chamomile or wormwood tea, to which some oil may be added, will often succeed admirably well. The sulphuret of potash may be used in the same way, or aloes dissolved in milk.

The tincture of the muriate of iron in water has been highly recommended by some writers; by others lime water is much esteemed.

M. *Martinet* advises that three different kinds of enemata should be administered, one after the other, at short intervals: first a common purgative injection to evacuate the bowels; then one to kill the worms and bring them away, consisting of common salt, or of vinegar with some bitter infusion; and lastly, an emollient oily one to soothe the irritation of the gut.

*Bremser* recommends an enema, consisting of the infusions of absinth, tansy, orange peel, and valerian, with a small portion of the empyreumatic oil of hartshorn, immediately after each alvine evacuation, as it is more likely then to be retained, and as it will also come more immediately in contact with the animalculæ.

Injections either of cold water, or of a few ounces of olive oil, to which several drops of laudanum or of hydrocyanic acid may be added, will generally relieve the irritation of the anus, which is often so distressing a symptom.

By some of these means, the worms may almost always be dislodged and removed. To prevent their reproduction, it is necessary that the use of a tonic aperient be continued for a considerable time. Pills, containing rhubarb, aloes, myrrh, and sulphate of iron, will answer very well in a number of cases.

A powder, consisting of rhubarb, worm seed (semina santonici) and carbonate of soda, is well suited for children. The patient should be recommended to use a good deal of salt with his food; and vegetables and fruit should on the whole be abstained from.

2. *Lumbrici.*—Stronger purgatives are necessary for the expulsion of these worms than of the ascarides. A powder, containing calomel, jalap, and rhubarb, is perhaps as good a formula as can be adopted. *Rosenstein* has recommended a combination of sulphate of iron, jalap, and wormseed powder and sugar, to be taken for three mornings successively; while *Stork's* favourite remedy was an electuary composed of sal polychrest, jalap, and valerian, a drachm of each, and four ounces of oxymel of squills—in doses of half an ounce three or four times a day to an adult.

*Bremser* tells us that he derived great benefit from a combination of wormseed, tansy, valerian, jalap, and sulphate of potash—an excellent formula, if the compound was not so nauseous. When the worms are expelled, he recommends a mixture containing tincture of aloes, steel, and elixir of vitriol.

The cowage (dolichos pruriens), granulated tin, and steel filings have occasionally been used, made into an electuary with syrup or treacle, with advantage; but of late years they have generally been superseded by other remedies. A combination of carbonate of iron, powdered wormseed, and scammony makes a good formula. Equal parts of infusion of senna and of the infusion of the spigelia, or of the decoction of pomegranate bark, may be taken at the same time with benefit.

We have not yet alluded to two of the most powerful anthelmintic purgatives—viz. croton oil, and the spirit of turpentine. The former has the great advantage of being so easily administered; even the external application of a few drops, rubbed on the abdomen, will occasionally succeed in dislodging the worms. The turpentine may be either given by the mouth or administered in an enema; the oil may be suspended by means of gum arabic in milk or gruel.

Common table salt has been highly recommended against lumbrici by the late celebrated physician, Dr. *Rush* of Philadelphia, in doses of half a drachm every morning before breakfast for a length of time. A glassful of sea water may be usefully substituted.

The tolerably free use of somewhat salted meat for food, at the same time avoiding fruits and vegetables, has often been observed to be attended with much benefit to persons subject to worms.

Friction of the abdomen with some liniment containing turpentine, ox-gall, absinth, aloes, and such like medicines, or the application of a plaster containing assafœtida, galbanum, camphor, rue, &c. are certainly sometimes useful.

3. *Tœnia.*—The spirit of turpentine has of late years almost quite superseded every other remedy for the expulsion of this kind of intestinal worm. In administering it, the physician must be especially attentive to secure its purgative action; otherwise the urinary organs are apt to suffer from excessive irritation. For this purpose the patient should always be instructed to take a full dose of castor oil, or of some other certain aperient, in the course of an hour or two after the turpentine has been swallowed, if the latter has not already acted on the bowels.

It is well known to cause in many instances vertigo and great disturbance of the head, amounting sometimes to a state of complete intoxication; but these symptoms will gradually subside. The essence of lemons is perhaps the best disguiser of the unpleasant taste of terebinthinate medicines. Chewing a piece of orange peel immediately afterwards will often relieve the nausea.

The pomegranate bark, either in the form of powder or of a strong decoction, has been successfully used by several medical men. One or two scruples of the powder in a wine-glassful of cold water is to be repeated every hour to the fourth, fifth, or sixth dose. If the worm is not expelled, the same medication is to be repeated on the following day. The decoction is the form in which it is generally used by the French physicians: it is prepared by boiling two ounces of the bark in a pint of water; a wine glassful is to be taken every half hour or so, till half the pint is swallowed. It is well to follow it up with a purgative, in case it does not act on the bowels.

Most of the remedies, which we have alluded to as useful against *lumbrici*, have been employed by different practitioners against tape-worms.

A combination of tin filings, fern root, wormseed, scammony, gamboge, and sal polychrest is a favourite remedy in some parts of Germany. Should the worm be partially extruded from the gut after a stool, the patient should continue to sit over the night-table, and swallow repeated doses of a solution of Epsom salts, or of any quickly-acting aperient, to induce further evacuations until the entire worm be expelled. Any attempts to pull it out with the fingers, or by affixing a portion of thread or tape to it, will almost always fail.

A course of vegetable and metallic tonics, with the addition of gentle aperients, should be continued for a length of time: the best are pills composed of aloes, rhubarb, and steel, along with some vegetable bitter infusion to which alkaline or neutral salts are added.

---

### On Epidemic Meningitis.

Our readers, says the *Gazette Medicale*, must have heard of the epidemic of meningitis which, during the last few months, has prevailed at Versailles, Rochfort, Metz, and more recently at Strasbourg.

Dr. *Wunschendorff* has published a narrative of the disease, as it was observed in the latter town. He tells us that, after having reigned exclusively among the soldiers of the garrison, the inhabitants of the town became affected. The first case observed at the clinique occurred on the 14th of January; then, after an interval of six weeks, new cases presented themselves successively up to the 1st of June, since which date no fresh case has been seen. During this period of time, four months and a half, 40 patients were admitted with the disease; and of this number 21 died.

The exciting *causes* of the epidemic remain quite inscrutable. In some cases, the disease appeared to spread by contagion; but the fact is not clearly made out.

The *necroscopic* appearances were those indicating intense inflammation, which frequently developed itself with a truly frightful rapidity; extensive injection of the pia mater, effusion of gelatiniform and somewhat purulent matter on the surface not only of the encephalon, but of the greater part of the spinal marrow. There were also, in almost all the cases, traces of a rudimentary or nascent inflammation of the villi and follicular glands of the intestines. Dr. *Wunschendorff* is therefore inclined to regard the disease of a *franchement inflammatoire* character. (If by this term the writer wishes to imply that the disease was a simple uncomplicated inflammation of the membranes of the brain, we have no doubt, *à priori,* that he is in error, and that he has allowed himself to be misled by the doctrines of the Broussaian school. In our opinion, every genuinely epidemic disease is necessarily dependent upon some atmospheric miasm, which induces a certain change in the circulating fluids, either by being directly introduced and so blended with them, or by some secret influence on the nervous system re-acting on the whole economy, and simultaneously affecting all the fluids and solids. This seems to us to be the only rational view that we can take of the development and diffusion of febrile diseases, which are not obviously the result of an antecedent local irritation, or of the suppression of an accustomed secretion, as of the perspiration or of the milk for example. The practical value of this view of epidemic disorders is, that the physician is thereby led to a more safe and judicious mode of treating them than that inculcated by *Broussais* and his disciples. The fever has a certain course to run before the system is able to throw off the morbific cause; and hence all attempts to strangle it by violent remedies is unscientific and dangerous.—*Rev.*)

The *symptoms* of this epidemic meningitis were usually sudden headache, vertigo, and vomiting; then delirium, convulsions, and tetanic spasms of the jaw and trunk; and lastly coma, paralysis of the sphincter and other parts of the body, and the usual phenomena of malignant typhus. The bowels were in most cases constipated at first, and subsequently became relaxed. The pulse was often remarkably slow at the commencement and in the early stage of the disease. In the majority of cases an herpetic eruption about the lips made its appearance.

According to the predominance of certain symptoms, Dr. *Wunschendorff* describes an inflammatory, a neuralgic, a convulsive, a delirious, and a comatose form of the disease. But he has not been able to point out a connection between these different forms and any special modifications of the necroscopic phenomena —on the contrary, the morbid lesions discovered on dissection were almost always very nearly the same.

The *course* of the disease was very irregular; in many cases it exploded with symptoms of high excitement, under which the patient sunk, or which were succeeded by torpor and various other phenomena.

The duration of the disease also varied much in different cases; in some it proved fatal in a few days, or even in a few hours, while in others it was lengthened, after the first violence passed away, to one or several months, before terminating in death or in a slow and painful convalescence.

The *diagnosis* was occasionally difficult, the disease assuming the characters either of malignant fever, follicular enteritis, or of apoplexy.

The *prognosis* was necessarily unfavourable. One half of the patients died; but this mortality is common to this epidemic with sporadic meningitis, which usually proves fatal in two of every three cases. In the civil hospital at Strasbourg, as we have already stated, 21 patients died out of 40 that were admitted; and in the military hospital the mortality was still greater—104 out of 176. The average age of the patients was from 18 to 25 years.

The *treatment* followed was actively antiphlogistic. The patients were usually bled several times, and at short intervals—varying according to the severity of the symptoms—during the early stage. Leeches were applied in great numbers to the temples and behind the ears, and cupping also along the spine was practised freely. Cold lotions were applied to the head, and in some cases mercurial ointment was smeared on the shaved scalp.

Having lost several patients, to whom calomel in large doses was administered, and as there were usually abdominal symptoms present, Professor *Forget* subsequently discontinued the use of intestinal derivatives, and trusted altogether to mild aperient enemata.

In the more advanced stage of the disease, much reliance was placed on cutaneous irritants. At this period, opium, in the form of syrup, seemed decidedly useful in mitigating the headache and delirium. All stimulant and tonic medicines were hurtful, except during the stage of convalescence.

We await anxiously the work which the Professor himself has announced to be in preparation, to learn more accurately the minute history of this very formidable epidemic.—*Gazette Medicale.*

---

### Spontaneous Combustion of the Human Body.

The following conclusions are drawn by M. *Jacobs* from the reports of 28 cases related by different authors.

1. Spontaneous combustion occurs only in human beings, and never in the lower animals: and among the former only in the living and never in the dead body.

2. The majority of cases occurred in persons advanced in years; the youngest was 29 years old, and there were other two 50 years old; all the rest were above the latter age.

3. By far the greater number of victims were women: of the 28 cases, two only occurred in men.

4. In one case the catastrophe was preceded by a state of jaundice; and in another by the existence of an unhealthy ulcer on the head.

5. All the persons were alone at the time of the accident.

6. They had all led an inactive life.

7. They were all very fat and corpulent; with the exception of three of the women, who were thin, and of a dry habit.

8. The majority, but not all, had been addicted to drinking spirituous liquors.

9. In most of the cases, there had been a candle, or fire, or some burning body close to the spot where the accident occurred.

10. Spontaneous combustion usually proceeds with great rapidity, as in from one to six or seven hours.

11. The flame proceeding from the body was not readily extinguished with water; it was very lambent and moveable, and destroyed only such articles as were quite close to, or in immediate contact with, the burning body.

12. The room, in which the combustion had taken place, was in most of the instances filled with a dense smoke, and the walls were covered with a carbonaceous substance; the floor, the bones, and the ashes were coated with an unctuous and highly-fetid matter.

13. The trunk of the body was generally quite destroyed; the remains being only some parts of the head and of the extremities.

14. All the cases of this singular occurrence, with the exception of two, have taken place when the weather was cold, and usually during Winter, and in northern regions.—*Casper's Wochenschrift.*

---

### On the Treatment of Diseases deemed incurable. By Professor Forget.

*Bacon,* in his celebrated work De Augm. Scient., uses these prophetic words: —*Neque igitur dubitabo inter desiderata reponere opus aliquod de curationibus morborum qui habentur pro insanibilibus.* We have not indeed the presumption to suggest any remedy or remedies for those diseases that are usually considered incurable: our only object is to draw the attention of medical men to a subject which well deserves their most serious reflection.

We do not propose to canvas the question what diseases are, and what are not, incurable, as this would lead us into a tedious and perhaps unsatisfactory discussion of terms; nor shall we venture at the present time to endeavour to explain how such diseases usually prove fatal to life.

In the case of such organs as are double, Nature herself sometimes supplies the deficiency caused by the destruction of one of them; we may allude, for example, to certain cases of the destruction of one kidney, or of one testicle, &c. The destruction of parts, which are not essential to life, can be viewed only as an infirmity. *Eh bien!* let us take the case of the most noble of all the viscera, the encephalon. Now it is an admitted axiom in cerebral pathology, that the destruction of those parts, which preside over certain nervous functions, causes the definitive abolition of these functions. Is this axiom true in all cases? Are there not examples of apoplexy, of softening having induced the melting down (*fonte*) of a part of the brain, and of protracted paralysis, which nevertheless have ultimately been cured? In propounding this axiom, have pathologists calculated all the resources which Nature can develop by means of nervous anastomoses, and all that may arise from following out a judicious and persevering course of medical treatment? Such fortunate results are rare, it is true; but that they do exist, cannot be disputed; and, despite the proverb, these exceptions, it must be admitted, really do invalidate the rule.

Now what is true of the destruction of organs, must, *a fortiori,* be still more so of the alteration of tissues. The greater number of organic lesions consist in the deposition of matter, which is either analogous or not to the natural tissue of the part. *Eh bien!* Nature possesses a power directly opposed to this tendency to abnormal secretions—viz. absorption: the difficulty consists in putting it into operation. Perhaps the mechanism, or *modus operandi,* of our alterative and discutient remedies only consists in the property of quickening this power of absorption.

As to the morbid states called functional or dynamic, in which there is no appreciable alteration of tissue, as we are ignorant of their essence, we have always a right to anticipate a cure of them. This remark is, we suppose, intended to apply to such diseases as tetanus and hydrophobia.

These considerations, which might be easily extended, are so many motives for encouragement to attempt the cure of some diseases that are still deemed incurable, although it must be confessed that we must not allow ourselves to be at all sanguine about the results, and we should ever be on guard against being misled by many statements which we every day hear of marvellous cures of such and such disorders.

*There* lies the difficulty of practice; for the more rebellious that a disease is, the more energy is required in attempting to subdue it, and the greater is the danger of active remedies causing the supervention of fresh accidents on the original malady. To know when to do nothing is a precept but little understood by the majority of medical practitioners, who seem to imagine that there is always a possible remedy for every form of disease, whatever that may be.

But listen on this subject to some of the oracles of our profession. *Frank* expressly tells us, "when a disease is recognised to be incurable, we ought to abstain from all vain efforts to try to cure it; and this precept is especially applicable to such therapeutic means as are unpleasant or painful. The medical man who employs such is no better than an executioner; it is better that a patient should sink under his disease, than that he should die from the effects of any remedies that are employed." *Sydenham* and *Stoll* have expressed the same sentiments; and *Tissot* very justly remarks, "It is the part of a skilful physician sometimes to prescribe no medicines at all."

*Pernicious Effects of powerful Remedies in Chronic Diseases.*—In truth, how many paralytic patients sink under some cerebro-spinal lesions excited by stimulating remedies of the nervous system, such as strychnine for example! How many phthisical patients fall a sacrifice to a colliquative diarrhœa, aggravated by the use of pretended incisive and solvent (fondants) medicines, as alkalis, which only serve to irritate the mucous membrane of the stomach and intestines! How many dropsical patients suffer from the use of drastic purgatives, or of powerful diuretics and diaphoretics, all of which must primarily act on the alimentary canal! How many unfortunates, labouring under some organic disease of the heart, or liver, or spleen, are poisoned by the injudicious administration of digitalis, mercury, iodine, &c.! and, lastly, do we not often see the system undermined by the continued use of arsenic and other irritants in hopeless cases of cancer and other incurable disorders!

It would be easy, says M. *Forget*, to extend the catalogue of this lugubrious litany, known only to the consciences of medical men. Much evil has resulted from that perfidious maxim of Celsus, "*Melius est anceps remedium adhibere quam nullum*:" there is much sounder wisdom in that which inculcates upon us, "*primo non nocere*," and in the advice of *Stoll*, "*Nil magni facias ex merâ hypothesi.*"

Some, we know, will object to these remarks which we have now made, and charge us with unnecessarily abridging the useful exertions of the physician. It is not the use, but the abuse, of remedies that we are condemning. Even in the case of hopelessly incurable diseases, much may be done by the judicious medical man in the way of soothing pain, calming mental distress, and sweetening and even prolonging life: such is one of the most holy duties of our noble profession.

When *Sydenham* said that without opium he would renounce practice altogether, he no doubt had in view the long and mournful catalogue of chronic maladies, the suffering from which may be so often mitigated by this and other analogous medicines. Let us learn then how to assuage the pains which we cannot prevent, and even when we fail in our best endeavours, let us learn how to console the sufferer.

As to the means best fitted to limit, if not arrest, the ravages of incurable diseases, they may be all deduced from an important law of pathology, which is far too little understood and acted upon; it is that many chronic maladies prove fatal by the complications which they call into action. Thus apoplexy often kills by the softening of the cerebral substance induced by the extravasated blood, which has been acting like a foreign body; pulmonary tubercles excite a subacute inflammatory action in the surrounding parenchymatous tissue; aneurism of the heart and hypertrophy of the liver, by the serous and sanguineous congestions which they give rise to; and cancer, by the exhaustion of the whole system, and by the cachexia brought on from the absorption of ichorous matter into the blood.

Without, therefore, vainly persisting in attempts to withdraw the cerebral coagulum, the removal of which can only be the slow work of Nature, let us strive to combat the congestion that is induced by this fatal thorn; without trying to melt away rebellious tubercles, it is better to check and dissipate the inflammation which exists around them; without endeavouring to modify the nutrition of an hypertrophied heart or liver, diminish the quantity of blood that passes through them, and draw the irritative process outwardly, and act gently on the various emunctories of the body; and without ever dreaming of extirpating an organ like the uterus, when corroded with cancer, support the system by mild food, and keep the surface of the ulcerated parts clean with anodyne washes.

Whatever be the disease that exists, soothe and mitigate pain; for this not only exhausts the frame, but inevitably aggravates the local mischief. While we pursue with prudence and modesty our sanatory labours, it sometimes happens that kind Nature crowns with unexpected success our submission to its mysterious decrees; in combating the auxiliaries, we sometimes will triumph over the principal enemy: in several cases the apparent cure of phthisis in our own practice has been the fruit of a treatment purely lenitive, and in no manner specific; in truth, chiefly of a mild regimen.

Much may unquestionably be often done by a judicious regimen: "*Abstinentiâ et quiete multi magni morbi curantur.........optimum medicamentum est cibus opportune datus*," says *Celsus;* and *Jos. Frank* confirms the opinion of the Roman: "dietetic regimen constitutes at once the most certain and the most copious source of therapeutics. A great number of diseases are in truth to be cured much more surely by the judicious selection of food and drink, by change of residence, amusement of the mind, &c. than by all our arsenal of medicines;" and how substantially true are these few words of the great English physician, *Sydenham;* "It has often occurred to me that in the treatment of diseases we go on too fast, and that we should leave much more to be done by nature."—*Gazette Medicale de Strasbourg, No.* 1.

---

### ON THE OBSCURITY OF THE SYMPTOMS OF PNEUMONIA IN OLD PEOPLE.

Professor *Cazeneuve* prefaces his remarks on this interesting subject with briefly reporting two cases in illustration.

*Case* 1.—Madame B., 82 years of age, and of a frail constitution, but whose health had been almost uniformly good, was, after exposure to a draught of cold air, rather suddenly seized with a shivering fit, followed by general heat. The feverish state continued next day, and as there was a marked exacerbation towards the afternoon, the physician who was called in considered the case to be one of ague, especially as this disease was prevalent at the time in the neighbourhood. Next day Dr. *Cazeneuve* saw the patient, and was inclined to take the same view as his confrere: there was no cough, nor any complaint of pain in the chest; and this absence of pulmonic distress induced him to omit ausculting the chest. On the fourth day, however, the old lady began to complain of a feeling of *malaise* at the lower part of the right side of the chest. The sound of percussion over this part was found to be duller than elsewhere, and a tolerably strong rhonchus and also a bronchial sound, but no crepitant rale, were heard on auscultation: the rest of the thorax gave out a vesicular respiratory murmur. There was no dyspnœa, nor any expectoration present; the general prostration was great; the pulse was 110 and tolerably full. Twelve ounces of blood were ordered to be taken from the arm; but, as the patient would not consent to the operation, 15 leeches were applied over the affected part instead. On the following day, the bronchial sound was louder than before, and the dulness on percussion was greater; still there was very little cough, and no expectoration. The bleeding was again recommended, and in the evening ten ounces were taken away; it soon became covered with a thick buffy coat: the night was restless, and the patient occasionally delirious. The bleeding was repeated to the same amount twice next day, and the blood was again found to be sizy each time. On the following (the seventh) day, the bronchial respiratory sound was less distinct, and was in part replaced by a light submucous rale over the lower part of the right lung: repeated doses of antimonial wine were given, and a Burgundy pitch plaster was applied over the affected part of the thorax on the eighth day, a fourth bleeding to the amount of ten ounces was practised, and still the blood was found to exhibit a buffy coat. In the course of a few days from this time, the patient was convalescent, although a light submucous rale continued to be perceptible over the base of the right lung.

*Case* 2.—A physician resident in Paris, 62 years of age, exhibited nearly the same train of phenomena as those now recorded to have been present in the preceding case. There was no cough or expectoration; only a slight aching was felt in the back and between the shoulders; the sound on percussion of this part was rather dull, while the rest of the chest was quite resonant; no crepitant rale was audible, but only a loud bronchial respiratory sound; the general prostration was great. All the symptoms were checked within 48 hours by three bleedings from the arm, and the application of a number of leeches to the chest.

In this case, the pneumonia was so masked and so difficult to be detected without the aid of percussion and auscultation, that a distinguished physician of the metropolis pronounced the case to be one of rheumatism of the back.

*General Remarks.*—It is to be kept in mind that the chest of an old person in health is usually very resonant on percussion, in consequence of the diminished thickness of the muscular and tegumentary coverings, the absence of fat, the ossification of the ribs, and the prolonged *sejour* of the air in the enlarged pulmonary vesicles. On the other hand, the vesicular respiratory murmur is usually feeble; it is no longer the soft silky sound of younger age, but has always more or less of a blowing character. Besides, the expiratory sound is more distinct than it used to be—in consequence probably of the dilatation of the vesicles, and the diminished elasticity of the lung causing it to subside less slowly than formerly during the act of expiration.

Keeping these circumstances in view, we shall now briefly notice the chief peculiarities in the two cases which we have recorded above: these peculiarities were the unusual resonance of the thoracic parietes, and the absence of the crepitant rale, as well as of cough, expectoration, dyspnœa, and pain.

1. As we have already explained, there is normally a greater resonance of the chest on percussion in old than in middle-aged persons: hence it requires a greater extent of hepatisation, or a larger amount of effused fluid within the chest, to cause a decided dulness of sound: very rarely indeed is the dulness so great in old people, as we not unfrequently meet with in younger patients.

2. As to the absence of the crepitant rale, which, it is well known, is usually described as the characteristic auscultatory sign of pneumonia, we have to remember that, for the production of this phenomenon, it is necessary that the air encounters a more or less viscid fluid in entering the pulmonary vesicles, and that these vesicles expel the admitted air with considerable force. The smaller these vesicles are, the greater will be the elasticity of the lungs, and the more readily will the rale be generated; and *vice versâ*. Now in old age the vesicles are irregular and dilated, and the elasticity of the lungs is considerably diminished: hence the dry crepitant rale is rare, and the respiratory sound is usually rough and bronchial.

It is known that M. *Cruveilhier* attaches more importance in the diagnosis of pneumonia to the presence of the bronchial *souffle* than to that of the crepitant rale. This may probably be owing to his having studied the disease chiefly among the aged inmates of the Saltpetriere, of which he is one of the physicians.

3. Again, the absence of dyspnœa is likely to mislead the practitioner: it is probably owing to the slowness and the diminished activity of the respiration, as we advance in years. Hence any impediment to the free admission of the air into the vesicles is attended with much less embarrassment in old people. The absence or infrequency of the cough and expectoration may be owing to the circumstance of the mucous membrane being but little affected, the inflammatory action being confined chiefly to the parenchymatous tissue of the lungs.

4. As to the production of pneumonia in our two patients, let us remember that, in old age, the tonicity of all the tissues being much diminished, the blood moves slowly in the vessels; the circulation is therefore easily embarrassed, and hence even from a slight cause the alterations which constitute a phlegmasia are generated. At first there seems to be a congestion in the most depending part of the affected lung, and in three or four days afterwards the symptoms of pneumonia supervene. These ideas recal to our mind the *Boerhaavian* theories which so long were prevalent, and which have probably been too much neglected since the time of *Haller* and *Bichat*. It must be confessed that, in studying many of the diseases of old age, and more especially pneumonia, we often meet with a confirmation of the opinions of the learned professor of Leyden.

5. The two cases, which we have briefly alluded to, shew that we must not be over timid in the use of bloodletting and other means of depletion, even in very old patients. The debility and prostration are very apt to impose on the inexperienced; but let it always be remembered that the morbid process is much more enfeebling than even the active adoption of antiphlogistic measures. Moreover, leeches or even cupping the chest is not nearly so useful in old as in middle-aged patients, in consequence of the diminished activity of the cutaneous vessels as we advance in years. As a general remark we may observe, that the efficacy of these local depletions is proportionate to the age of the patient: hence their great utility in the treatment of the diseases of children, and their comparative powerlessness in the diseases of adult and of old age. We must, as a matter of course, be more guarded in the use of vigorous means in the treatment of old persons; but their mere age can never be a sufficient reason for not having recourse to bloodletting, whenever there is reason to believe the existence of inflammatory action in any important organ.

Whenever there is great prostration and feverishness at the same time in an old person, it is prudent to examine attentively the state of the lungs. The epidemics of adynamic fever observed by *Pinel* at the Saltpetriere were only so many cases of pneumonia among the aged inmates of this immense hospital. *Pinel* had not learned to attribute to the lesion of one organ this *ensemble* of symptoms which he described under the term of adynamic fever.* The adynamic state, which is so common in the acute diseases of old age, is attributable

---

* Dr. *Cazeneuve* surely pushes his views a little too far in this assertion; it is to be noticed that he is quite a supporter of the doctrine that refers all fevers to a local morbid lesion—a doctrine to which we cannot give our assent.—*Rev.*

to the difficulty experienced by the system at this period of life to repair the losses sustained (les pertes eprouvées) by the nervous system.

In conclusion, we may observe, that almost all the cases of pneumonia in old people may be traced to the influence of exposure to cold. Hence the importance of warm clothing, warm rooms, &c. for such patients. The great age of *Buffon* and also of *Voltaire* was probably owing in not a little degree to the extreme care which these celebrated men paid to the circumstances now mentioned. —*Gazette Medicale.*

---

### On Paracentesis Thoracis. By M. Sedillot.—Case of Paracentesis of the Pericardium.

The diagnosis of effusions into the cavity of the pleura is one of the most valuable discoveries of modern medical science; we need scarcely add that we owe it almost entirely to the aid of auscultation. We have only to read the works of *Baglivi, Morgagni,* and *Bayle,* (Recherch. sur la Phthisie, p. 335,) to discover how often, nay how generally, the nature of the disease in question was mistaken until revealed by dissection. When percussion first began to be practised, all was uncertainty and doubt. By means of this simple test, an important step in diagnosis was obtained: "*Verum si media pars aquâ repleta fuerit, evocabitur resonantia major in illâ parte quam aquosus humor non occupaverit,*" says *Avenbrugger,* in his truly valuable work. But with candour he admits the insufficiency of percussion on too many occasions. How pregnant with important truth are these words, in which he describes the insidious progress of effusions into the cavity of the pleura: "*Observavi sæpe sub tussi prope nullâ, aut respirationis incommodo, integrum thoracis latus sonitu carnisse tunc quando, ex febri acutâ convalescens, sub febri erraticâ vix ægrotare visus est medico, donec, morbo sensim crescente (forsan etiam tunc nondum cognitâ mali sede), vel hydropico tumore semisepultus, vel ad pellem et ossa usque consumptus, periisset.*"—(Invent. Nov. &c. Vindobonæ, 1761.)

........ Although great progress has been made of late years in the diagnosis of pleuritic effusions, it must nevertheless be confessed that the success of our treatment is on the whole far from being satisfactory. Hitherto the use of internal remedies has almost always failed in promoting the absorption of the fluid, when the quantity of this is at all considerable; hence we have been obliged to have recourse to a surgical operation to give the patient a chance of recovery. If this be not adopted, the patient becomes more and more emaciated, anasarca comes on, attended often with a colliquative diarrhœa and hectic fever, and, after a period more or less protracted according to the strength of the constitution, and still more to the nature of the effused fluid, whether it be purulent or not, death inevitably follows.

In the valuable thesis of M. *Raymond Faure,* recently published, we find the following directions as to the proper time to puncture the thorax. "It appears to us to be called for whenever a fair trial of those means that are calculated to promote absorption has been made, and without avail, for the relief of the symptoms; to insist too long on the employment of these curative means, the action of which is at best indirect and doubtful, appears to be not only useless but positively dangerous, for the following reasons: 1, the accumulation of the fluid within the pleura gradually increases the compression of the lung, until it ultimately prevents its expansion; 2, the fluid keeps up a constant irritation of the pleural membrane, either by distending it or by acting as a direct stimulant on its surface; 3, by separating the ribs from each other, it forms a mechanical obstacle to the obliteration of the costo-pulmonary cavity, which is the very principle and condition of a cure being effected; 4, the activity of the process of absorption is always inversely proportionate to the quantity of fluid effused; 5, the successive evacuations imitate the process of Nature, by which she sometimes effects a cure of empyema by forming an outward abscess in the thoracic parietes; and, 6th, the increase and prolonged *sejour* of the fluid in the chest brings on a cachexia of the whole system, which inevitably proves fatal in the long run."

M. *Sedillot* has taken great pains to collect the results of almost all the cases on record, and, while admitting that the success on the whole of the operation of puncturing the thorax has been far from encouraging, he points out with much justice that not a few of the failures have been attributable to an ill-judged delay, or to some other mismanagement of the case.

If we were to judge of the propriety of the operation from the practice of *Dupuytren*, we must entirely abandon it; for out of 50 cases, 48 proved fatal; and M. *Velpeau* tell us that of 12 cases, in which he performed it, not one was fortunate.

The directions which M. *Sedillot* gives are these: 1, not to operate during the acute stage of the disease; the rash trials made a short time ago at the Hotel Dieu prove the necessity of such an advice; 2, to operate, before the lungs have lost their power of expansion; 3, to put off the operation as long as possible, whenever there exist any incurable complications; for then it can only be regarded as a palliative.

Our author is however surely in error, when he states that the complete absence of all respiratory murmur is an absolute contra-indication of the operation. We have met with cases where no murmur could be heard at any part of one side, except perhaps in the subclavicular region, and which yet recovered ultimately. We are not therefore to abandon such cases as incurable, however unfavourable our prognosis may be; and, on the other hand, we must not be too sanguine as to the lung recovering its entire expansibility, merely because the murmur is distinctly perceptible. We need scarcely add that, whenever we have reason to suspect the complication of tuberculous deposit, our prognosis as to the result will necessarily be more guarded than otherwise.

M. *Sedillot* thinks that the nature of the effused fluid may be predicated from the existing symptoms; for there is always more fever and more local uneasiness according as this is more or less purulent. To a certain extent only this opinion is correct; but certainly no judicious physician will be too confident on this subject. As to his advice to try the effects of injecting any fluid into the cavity of the pleura, after withdrawing the effused fluid, we must positively and *in toto* condemn it as most dangerous, in spite of the high authority of M. *Recamier* to the contrary.

In the 33d vol. of the *Medicinische Jahrbucher*, recently published, there is a paper by Dr. *Schuh* of Vienna on paracentesis of the pleura, and also of the pericardium. During last year he performed the operation of puncturing the pleura in 44 cases—in eight of which a complete cure was effected, in four the patients died, and in the remaining 32, a decided, but not a permanent, relief was obtained. The chief danger consequent on the operation is the introduction of a considerable quantity of air into the cavity of the chest, when the fluid is withdrawn. This is apt to take place, it is well known, whenever a deep inspiration takes place.* He advises that a portion only of the effused fluid should be allowed to escape, when the disease has been of long standing: otherwise the lungs may not expand sufficiently to fill up the empty space. In some cases of

* Perhaps the simplest as well as the most effectual means of preventing this dangerous accident is that recommended by Dr. *Reybard* of Lyons, and described in the last number of the *Medico-Chirurgical Review*, p. 193.

purulent effusion, it is found expedient to retain a small caoutchouc canula in the wound, and allow the matter to drain off once or twice a day.

*Paracentesis of the Pericardium.*

In one case, occurring in a woman 24 years of age—in whom were all the symptoms, well characterised, of dropsy of the pericardium, to such an extent as to threaten suffocation—Dr. *Schuh* introduced a trocar between the third and fourth ribs, very near to the edge of the sternum, between it and the course of the internal mammary artery. At first only a few drops of blood flowed out: a small bougie, passed along the canula, touched the great bloodvessels, the pulsations of which were distinctly felt. The operation was immediately repeated between the fourth and the fifth ribs, when there flowed out slowly, and in a stream (en nappe), a certain quantity of reddish serosity. The patient slept tolerably well the first night, the dyspnœa being much less than it had been for a length of time, and, in a few days after, the general œdema of the legs was considerably diminished. By the end of the third week the effusion into the pericardium had quite disappeared.—*Gazette Medicale.*

*Remarks.*—Our chief reason for introducing the above notice of M. *Sedillot's* memoir is to draw the attention of our readers to a most valuable paper on the treatment of pleuritic effusion by the late Dr. *Hope,* whose recent loss must be deeply deplored by all who take an interest in thoracic pathology. He there points out how much may be done by a vigorous adoption of appropriate treatment; his chief remedies being the internal and external use of mercury, the application of repeated blisters, the administration of diuretics, and occasionally of hydragogue cathartics, and the use of a nutritious animal diet to support the strength of the patient. His remarks on the errors of diagnosis of this complaint are especially important, and likewise his novel observations on the means of arresting the hectic fever which is always present, when the effused fluid is purulent. According to his extensive experience, it is rarely necessary to have recourse to the operation of puncturing the chest. "If," says he, "Dr. *Stokes* has cured twenty cases running by Lugol's solution and ointment of iodine, together with blisters and other means, and I have cured thirty-three consecutive cases by other means, we must admit that fifty three cases cured successively, without selection, afford a strong presumption that all really *curable* cases are curable without paracentesis." This announcement is at once most unexpected and most gratifying. The whole of Dr. *Hope's* paper cannot fail to be read with the deepest interest by all medical men.—*Rev.*

---

### Consultation on a Case of Hermaphrodism.

M. *Benoit* was consulted last year by Marie B——, 27 years of age, and registered in one of the towns of the department of Tarn as a girl.

When 14 years old she became aware that there was some peculiarity in the conformation of her genital organs, and she therefore applied to a medical man, who assured her parents that a simple operation would set her all right. An incision was made for the purpose of re-establishing the opening of the vagina; but this not being found, nothing else was done. Shortly before consulting M. *Benoit* she had been asked in marriage, and at first refused the offer; but, as her lover persisted in his addresses, she at length declared the cause. The account, which she gave to M. *Benoit,* was as follows: when about 14 years of age, she observed in the right inguinal region a small swelling, which was very painful for some time, but disappeared soon afterwards. Although she suffered considerably from headache, languor and general uneasiness, there had never been any sanguineous discharge....... Below the mons veneris, which is altogether similar to that usually observed in women, there is a fissure terminated on each side by two thin corrugated labia, which are slightly covered with hair, and stretches backwards towards the anus. This fissure is only about three quarters of an inch deep: its anterior commissure terminates in a diminutive penis-like organ, which is provided with a prepuce, a gland, two cavernous bodies, but without any terminal aperture; it is subject to erections, and becomes then considerably longer. At the root of this organ, on its inferior surface, there is an opening which communicates with the urethra; constituting therefore the *vitium conformationis* known by the name of hypospadias. This opening, at rather less than three inches from the anus, leads into the bladder by a canal which is curved, as in the male subject; but it is only about three inches long. When a sound is introduced into the bladder, and a finger passed up the rectum, there seems to be only a thin septum between them, but not any trace of prostate gland. In the inguinal region (it is not stated on which side) there is a small round and moveable body under the integuments.

From this state of parts, M. *Benoit* gave it as his opinion that the sex was male, and that his patient was incapable of contracting marriage.—*Journal de Montpelier.*

---

### M. RACIBORSKI ON THE PHYSIOLOGY OF MENSTRUATION.

Our readers will remember that in our recent review of *Gendrin's* Traité Philosophique, we drew their attention to this curious, and hitherto ill-understood, subject of physiological enquiry. From the researches of this gentleman, and from those of M. *Negrier*, as well as of Dr. *Robert Lee*, it was suggested that there is an actual rupture of one of the ovarian vesicles at each period of menstruation, and that the sanguineous discharge from the uterus was the result of this lesion. M. *Raciborski* questions the accuracy of this statement. While he admits that the primary movement in each act of menstruation is a congestion of the vessels of the ovaries, he denies that any rupture of their surface necessarily takes place at the same time.

"Having examined," says he, "in a great number of cases, the ovaries of women who had borne children, we feel assured that, as a general rule, the number of the cicatrices on the surface of the ovaries is always proportionate to the frequency of actual impregnation, whether it has been a genuine or only a false conception."

Some of the subsequent statements of M. *Raciborski* himself seem, however, to be at variance with this assertion, and partially to confirm the idea of frequent, if not of invariable, rupture during menstruation. Having quoted the opinion of M. *Negrier*, which is expressed in the following words: "An afflux of transparent fluid takes place into the cavity of one of the superficial vesicles of the ovary: this fluid, by its accumulation, depresses the yellow matter, distends and attenuates this last (the vesicle?) at the point which presents the least resistance; the ovarian envelopes are at length raised up, distended, and ruptured, with the vesicle," our author thus comments upon it.

"According to the distinguished professor of Angers, there must take place, once a month, in the female constitution, a phenomenon analogous to what we observe to occur in many birds. Women, like hens, must have the power of detaching ova from their ovaries without any previous fecundation. But, before we can admit so startling a proposition, we require to have more conclusive data than those hitherto made public. As to the statement that cicatrices with red edges, or small pouches filled with blood, have been found in the ovaries of women who had menstruated shortly before death, we may observe that on many occasions we have observed similar alterations in the bodies of women in whom the catamenia had been suppressed for several months, as is generally the case, for example, in those who die of phthisis."

M. *Raciborski* sums up the conclusions to which he has come, after a very elaborate enquiry, in the following propositions:—

1. That menstruation is a consequence of the accomplishment of the development of the ovaries.

2. That it is the direct result of the means employed by nature to place the ends of the Fallopian tubes and the ovaries in the relations necessary to fecundation and the passage of the fecundated ova.

3. That the sanguineous congestion, which is indispensable for obtaining those conditions in the human being, appears sufficient in itself to explain the occurrence of the hæmorrhage which constitutes menstruation—without having recourse to supposing that there is any necessary solution of continuity.

5. That the vertical position, favouring still more the effects of sanguineous congestion on the generative organs, may be one of the principal reasons of the abundance of the menstrual flux in women, and in some species of simiæ.

5. That, for want of having precise information as to the nature and theory of menstruation, it has been hitherto impossible to establish a rational treatment of the various disorders induced by irregularities of this function.

6. That it is not yet sufficiently proved that the ovula arrive successively to maturity at each menstrual epoch, or that the most mature ovum then approaches nearest the surface of the ovarium, there to become ruptured and give exit to a germ.—*L'Experience.*

---

### M. Magendie on Animal Magnetism.

At the meeting of the Academy of Sciences on the 24th of June last, M. *Magendie* read a report on a case of alleged cure of a deaf and dumb person, which had been communicated by M. *Dupotet* (the Baron, we suppose, who took French leave from London two or three years ago).

As we had not seen the patient, says the reporter, before she was under treatment, we could not have vouched for the cure, even had it been complete; but unfortunately for both patient and doctor, it was far from being so. The case was simply one of deaf and dumb infirmity, in which the invalid could perceive sounds when very loud, and could understand a few articulate words.

To judge of the efficacy of his treatment, we proposed to M. *Dupotet* to try it on three inmates of the deaf and dumb institution, whose actual state might be ascertained beforehand.

He agreed to this proposal, and required eight days for effecting a cure: we granted him fifteen. The three persons, whom we entrusted to his care, were not insensible to every kind of sound,—in truth, this extreme degree of the infirmity is very rare—all of them could recognize certain sounds, if not by the ear, at least by the vibrations of surrounding bodies. At the end of the eight days, M. *Dupotet* represented them as cured, and as capable, with proper instruction, of taking that position in society to which they were entitled. Our three patients had, we admit, gained a little more sensibility of hearing; the explosive words, such as *papa, Dupotet,* were pronounced with rather more facility than formerly: but this was all the extent of the improvement.

They were not at all surprised themselves at the change, and the physician of the establishment told us that the amendment was in no respect different from what might at any time be effected, whenever the faculty of hearing is more than usually exercised, but that this continues for a very short time, and then the patients relapse into their former condition.

M. *Magendie* stated that he had recently seen the deaf and dumb boy, Tresel, who had been said to be cured by injections into the tympanum of the ear. Although the case is now of long standing, and he has received all the benefit of a most assiduous care, his speech is still extremely imperfect, and he can scarcely do more than repeat the words which he is in the habit of hearing pronounced in his presence. It seems therefore that it is not sufficient to restore to deaf and dumb persons the power of hearing and speaking, but that it is necessary to give them at the same time the instinctive desire of speech, which induces children to practise it of themselves, without being solicited.

As to the three patients committed to M. *Dupotet*, they have not continued the treatment which he used, although an offer was made to him to send them to his house three times a week for the purpose. Since that time, we have heard no more from him. We must therefore in conclusion pronounce that the cure alleged by him to have been effected by animal magnetism is quite without foundation.

It appears from the report of a subsequent meeting of the Academicians, that it was resolved by a large majority, that they will not hear anything more on the subject of this mysterious science! Several of the members indeed objected to this peremptory decision, asserting that, although there is much quackery in many of the statements of its admirers, there seem to be some observations and phenomena, which deserve to be more attentively examined. MM. *Cloquet* and *Bouillaud* expressed themselves to this effect; and on the other hand M. *Breschet* said that, as the Academy of Sciences had come to a resolution to proceed at once to the order of the day whenever any question respecting the quadrature of the circle or perpetual motion was brought forward, the subject of animal magnetism should be dealt by them in the same way.

---

### Annual Report of the Vaccination Committee of France.

The following are the general conclusions of this Report read by M. *Gauthier de Claubry*, at the Royal Academy of Medicine.

1. In the epidemics of small-pox which have occurred in different departments, vaccination has unquestionably continued to possess the power of arresting the evil, by reducing the genuine variola to a varioloid disease.

2. Throughout France the immense majority of those who have been some time vaccinated has remained unaffected either by the sporadic or by the epidemic variola, although in immediate contact with those who were suffering.

3. Vaccination invariably induced a favourable modification on the course of variola, whether the disease was in its early or in its advanced stage.

4. Some cases of varioloid disease which nearly resembled variola, and several even of variola itself, have occurred in vaccinated persons, although many of the observations are manifestly incorrect. For, on the one hand, the appellation of variola has often been given to an eruption which has lasted only three days; and, on the other hand, it is but too apparent that many of the certificates of an alleged successful vaccination cannot be trusted to. We can confidently affirm that, after an attentive examination of numerous cases of variola and of varioloid eruption occurring after vaccination, we have always found the disease to be less severe than eruptions of the same nature appearing after a first attack of variola.

5. The majority of observers deny that the vaccine virus has undergone any change by having been transmitted through a multitude of persons. Several practitioners have vaccinated one arm with the ancient, and another arm with the renewed, virus, and the vesicles on both arms have been observed to be quite the same.

6. Most medical men disapprove of re-vaccination, at least as a general measure, on the ground that, by thus exhibiting a want of faith in the protecting powers of the remedy, the confidence of the people would be inevitably much shaken, and greater difficulty than hitherto would be experienced in inducing them to submit to it. Moreover, the partisans of re-vaccination are not agreed as to the proper time of resorting to it.

7. In 1839, of 6652 re-vaccinations, which were carefully watched, a pustule similar to the normal one was observed in 718 cases; in 1283 cases the pustule was of a doubtful character; and in 4651 the operation failed completely. It thus appears that a large majority of persons are not capable of contracting vaccinia twice, and consequently that re-vaccination is generally useless.

8. Even when re-vaccination has had a positive result, it has not always protected the person from the contagion of variola.

9. Even supposing that re-vaccination had the effect of bestowing complete protection, we could not in this way hope to extinguish the variolous infection, as it would be almost impossible to cause it to be adopted as a universal measure.

M. *Piorry* observed that he heard with regret the statement in the preceding report, that re-vaccination should not be adopted as a general measure. I have, said he, practised re-vaccination in a great number of cases, and, without having kept an exact account of the cases where it produced a positive result, I may confidently affirm that, in a fourth or fifth of the cases, the operation has induced a local eruption, the characters of which are to those of genuine vaccinia as the characters of the varioloid disease are to those of genuine variola. Is it not, therefore, possible that re-vaccination might protect from the varioloid disease, which we know is sometimes very severe, as the first vaccination does from variola itself? For this reason I am of opinion that some of the conclusions of the report be not so decided and peremptory as they at present stand.

M. *Bousquet*:—From the trials of re-vaccination, of which I have been able to verify the results, it follows that, of 138 operations, a perfect normal pustule was induced in 30. I re-vaccinated 90 persons at Versailles, and of this number I obtained 37 genuine vaccine pustules. These results are conclusive, in my opinion, in favour of re-vaccination; the facts cannot be disputed, for they occurred under the inspection of two excellent practitioners at Versailles, MM. *Vitry* and *Boucher*.

Several other members of the Academy, including M. *Bouillaud* and M. *Dubois*, agreed with the preceding speaker, that the report of the committee was too decided and peremptory in some of its conclusions.

*Remark.*—It would seem that the results of re-vaccination are very different in different countries. Nowhere has it been practised with so much success and on so large a scale as in some parts of Germany. We observe that, during last year, 42,522 soldiers in the Prussian army were re-vaccinated; in 34,573 of whom, distinct cicatrices on the arm were present, in 6177 the cicatrices were indistinct, and in 2772 none were to be seen. The progress of the re-vaccination is stated to have been regular in 20,952 persons, irregular in 8,820, and to have failed in 13,750; in several of the last the operation was repeated, and with success. The more that we consider this important question, the more convinced are we that many statistical reports are little to be trusted to, as affording safe or conclusive data to build general deductions upon. Unfortunately a great number of first vaccinations are reported to have been successful, without having been properly examined.—*Rev.*

### SCENE IN THE FRENCH ACADEMY.

M. *Amussat* read a new communication on the cure of stammering by a surgical operation. Having stated that up to the present time (25th February) he had performed the operation of dividing the *genio-hyo-glossi* muscles in nine cases, he now brought forward two of the patients to be examined by the members, and other two on whom he proposed to perform the operation, in order that no doubt might exist as to the extent of the infirmity to be relieved. He then exhibited to the Academy a pathological specimen, which he had received from M. *Begin;* this was the tongue of an old soldier, who had always stammered a great deal in his speech, and had recently died at the Val de Grace. The two lateral halves of the organ were of unequal size, the left one being flattened, while the right was thick and swollen. This state of the tongue seems to shew that one of the chief impediments to unembarrassed pronunciation consists in an abnormal conformation of the organ; being too short or drawn to a side (*devié*) by the contraction of the genio-glossi muscles, their section will account for the relief given by the operation.

M. *Velpeau:*—I must confess that the examination of stammering persons, as usually practised by the Academy, offers no serious guarantee for truth. We all know that the infirmity varies a great deal from a number of influences. It would be more rational, in my opinion, to submit all the patients to an attentive examination by a committee appointed for the purpose.

M. *Dubois* concurred in the sentiments now expressed by M. *Velpeau.* He did not think it consistent with the dignity of the Academy to see patients mounted up upon the desk, as if it was a shew-place, and made to repeat the same words and answer the same questions, like parrots.

Here M. *Amussat* rose to call the member to order for the offensive expressions he was using. M. *Dubois* however persisted, and expressed his surprise at the reclamation of the learned gentlemen.

At this moment, M. *Gerdy* called upon the president not to open a discussion, except when the patients have been sufficiently examined, and have left the hall. Some members having called upon M. *Amussat* to explain the processes which he followed in performing his operations, the president, M. *Roux,* availed himself of the opportunity to state, that a few days ago he divided the *genio glossi* muscles in a stammerer. The patient, immediately after the operation, pronounced some words with rather greater facility; but he has since refused to speak at all, and, as considerable inflammation came on in the mouth, a number of leeches have been applied.

M. *Amussat* here was proceeding to explain his operation, when M. *Gerdy* again rose and said that he had possession of the chair: he declared that a most rigid examination of the alleged cures should be instituted by a special committee. The operation, he went on to say, exhibits two sides, one moral, the other scientific; hitherto however he could only see a commercial (industriel) side in any of them. This word excited among the learned academicians a storm which it defies all language to describe. Amid the most dreadful shoutings, violent apostrophes, and continual interruptions, M. *Gerdy* insisted upon reading from a provincial journal an article in which the operations of M. *Amussat* and of M. *Boyer* are described in the most pompous terms. During the reading of this paper, M. *Amussat,* the prey of the most lively agitation, was continually repeating, in the most solemn terms, his entire ignorance of the article in question. All the academicians rushed into the *hemicycle,* forming a ring round M. *Gerdy;* some calling upon him to continue, others to stop, while many of the members at the same time were shouting to the president to dissolve the meeting. After having made some ineffectual attempts to restore order, M. *Roux* declared that the meeting was dissolved.

"It is utterly impossible," says the reporter, "to give any just idea of the extreme confusion at the close of this meeting of *savans;** it recalled to our minds the recollection of those stormy political assemblies, which are faithfully recorded in the past history of our country."

(So much for the deliberations of the Royal Academy of Medicine in the metropolis of the most polite people of the world!)

---

### M. Dubois on the Signs of Pregnancy.

In treating of the signs of pregnancy furnished by the state of the mammæ, M. *Dubois* says—

"In a pregnant woman, the puffy swelling of the areola is a sign to which Professor *Hamilton* of Edinburgh attaches the greatest importance, because it is, according to his experience, never to be observed in those who have not been exposed to conception. We observe indeed occasionally in maiden women some unusual appearances in the areola, but never the genuine *boursoufflement*. The accuracy of this opinion has been questioned by several physicians; but I must acknowledge that I am quite inclined to agree with Dr. Hamilton. Unfortunately the character of the areola to which we allude is far from being uniform or constant; but, when it is present, we regard it as one of the most trustworthy of the signs of pregnancy."

He afterwards remarks:—"The areola in a pregnant woman assumes a darker colour; it exhibits minute papillary eminences, which existed indeed beforehand, but which become more distinct and elevated; and in some cases it is raised up, prominent, and as it were œdematous. Around this first areola there is usually noticed a second one, which exhibits a spotted appearance. The veins of the mamma also are much larger than before. The elevation of the areola, now described, is unfortunately rather a rare occurrence; for it is a very important one, and almost infallibly indicates the condition of pregnancy."

*The Discolouration of the Areola.*—There is much discrepancy of opinion among accoucheurs as to the value of this sign; some regarding it as very characteristic, while others attach but little importance to it. The anecdote of Dr. William Hunter on one occasion pronouncing, from the presence of this sign in a female subject brought for dissection, that she was pregnant, is well known. M. *Dubois* acknowledges that the sign is a valuable one; for it is certainly present in a very great number of pregnant women; but he reminds his readers that its value is considerably diminished for the following reasons:—very frequently it is not observed at all, or it is very indistinct, in women of a fair complexion; the dark colour almost always remains more or less decided, when once a woman has conceived; and, thirdly, in certain uterine diseases the areola is apt to be discoloured, although the woman has never been exposed to the chance of impregnation. He adds: "Each of the signs now mentioned—the discolouration of the areola, the secretion of milk, the prominence of the papillæ, and the

---

* While reading this, we were involuntarily reminded of the witty anecdote related by Las Casas in his memoirs of Napoleon. During the French campaign in Egypt, the *savans* and draughtsmen who accompanied the army were huddled with the baggage into the middle of the squares, the moment that the enemy appeared. No sooner were the Mameluke horse descried, than the word was given, "form square; artillery to the angles; asses and *savans* to the centre"—a command which afforded no small merriment to the soldiers, and made them call the asses *demi-savans*.—*Rev.*

puffy elevation of the areola—taken *separately*, cannot be well trusted to in any case; except perhaps the last, and this unfortunately is only of occasional occurrence. But when they are co-existent in the same case, they will generally be found sufficient to determine the presence of pregnancy."

M. *Dubois* makes repeated allusion to the recent researches of Dr. *Montgomery* of *Dublin;* but, while commending them for their accuracy in the main, he is inclined to believe that he attaches rather too great importance to some of the signs furnished by the state of the mammæ. He professes his inability to give any opinion as to the value of the sign that has been drawn from a change in the colour of the lining membrane of the vagina. "It has been alleged that when impregnation takes place, the surface of the vagina assumes a peculiar purplish or violet-blue colour, which seems to be the result of sanguineous congestion in the part. This discolouration is said to commence in the second month of pregnancy. It is considered by some writers as a certain sign of pregnancy, provided no hæmorrhoidal disease be present at the time; for this also is said to induce a change in the colour of the vaginal mucous membrane. M. *Jacquemin*, physician of La Force prison, in which there is always a great number of prostitutes, has stated in a work upon this subject, that he has very frequently observed the discolouration, and that he has always found it dependent upon pregnancy. His observations have been made on between four and five thousand women; and in no one instance has he been mistaken in his diagnosis, when this sign was present. As a matter of course it requires the speculum to be used to detect it properly. M. *Dubois* confesses that he can say nothing in its favour from the results of his own experience. It is to be remembered that it is admitted by M. *Jacquemin* himself, as well as by others who have faith in it, that the vaginal membrane may acquire a darker colour than usual from other causes besides pregnancy. If a woman be examined with a speculum immediately after the cessation of the catamenia, the cervix uteri and the vagina will be observed to be more or less congested. It is well known that, during the season of rutting in animals, there is always a turgescence of the generative organs. Whatever, in short, tends to cause a more than usual flow of blood to these parts will probably induce some change in the colour of the lining membrane of the vagina."

The attention of obstetrical practitioners has of late years been directed by several writers to the state of the urine during pregnancy. Dr. *Golding Bird* describes the peculiarities which the secretion exhibits in the following terms. If put aside in a glass vessel for two days, it was observed to become much troubled; innumerable globules, having a greasy or fatty aspect, appeared on its surface: after another day or two, it became completely covered with a pellicle, resembling that which forms on mutton broth when cooling; and, on the sixth day, this crust broke up and fell to the bottom. These appearances were observed in nearly thirty cases. Submitted to the microscope, the pellicle exhibited beautiful triangular prisms of triple phosphate of magnesia, contained in a mass of granular matter, and here and there patches of tolerably regular globules. Dr. *Bird* has observed the phenomena, now described, in the urine as early as the second month of pregnancy; and he assures us that he has derived great assistance in the diagnosis of pregnancy from attending to them. It will be very interesting if his observations are confirmed by the researches of others.—*—Gazette des Hôpitaux.*

